# Ultrasound-assisted visible-light photo-Fenton degradation of tetracycline over surface-engineered Ag/MgFe_2_O_4_ heterointerfaces: mechanistic and DFT insights^☆^

**DOI:** 10.1016/j.ultsonch.2026.108011

**Published:** 2026-08-18

**Authors:** Velu Subash, Duraisamy Elango, Palaniyappan Jayanthi, Velu Manikandan, Kwang Soup Song

**Affiliations:** a Department of Environmental Science, Periyar University, Salem 636011, Tamil Nadu, India; b Department of Biomedical Engineering, Kumoh National Institute of Technology, Gumi, South Korea; c High-tech Medical Equipment Research Institute, Kumoh National Institute of Technology, Gumi, South Korea

**Keywords:** Ag(3 wt%)/MFO, Surface engineering, Ultrasound-assisted photo-Fenton, TCH degradation, Density functional theory

## Abstract

The integration of ultrasound with visible-light photo-Fenton catalysis provides an attractive route for accelerating the degradation of persistent antibiotic pollutants. In this work, pristine MgFe_2_O_4_ (MFO) together with surface-engineered Zr(3 wt%)/MFO, Co(3 wt%)/MFO, and Ag(3 wt%)/MFO photocatalysts were synthesized to investigate the influence of surface engineering on tetracycline (TCH) degradation under ultrasound-assisted visible-light (US/VL) photo-Fenton conditions. Structural, morphological, and surface analyses confirmed that the deposited metal species formed stable heterointerfaces while preserving the spinel MFO framework. Among the prepared photocatalysts, Ag(3 wt%)/MFO exhibited the highest activity, achieving 98.4 % TCH degradation within 120 min under the optimized reaction conditions. The enhanced performance originates from improved interfacial charge migration, efficient H_2_O_2_ activation, and accelerated Fe^2+^/Fe^3+^ redox cycling under the combined action of ultrasonic cavitation and VL irradiation, resulting in enhanced ROS generation. Radical quenching experiments identified •OH radicals as the dominant oxidative species, while •O_2_^−^ radicals and photogenerated electrons contributed synergistically to the degradation process. The optimized photocatalyst maintained over 94 % degradation efficiency after four consecutive cycles, while post-reaction XRD and XPS analyses confirmed excellent structural and chemical stability. LC-MS analysis identified the degradation intermediates of TCH, while DFT calculations revealed the preferential ROS attack sites, supporting the proposed degradation mechanism. Overall, this work establishes surface-engineered Ag(3 wt%)/MFO as an efficient US/VL-photo-Fenton photocatalyst for sustainable wastewater remediation.

## Graphical abstract

**Figure:**
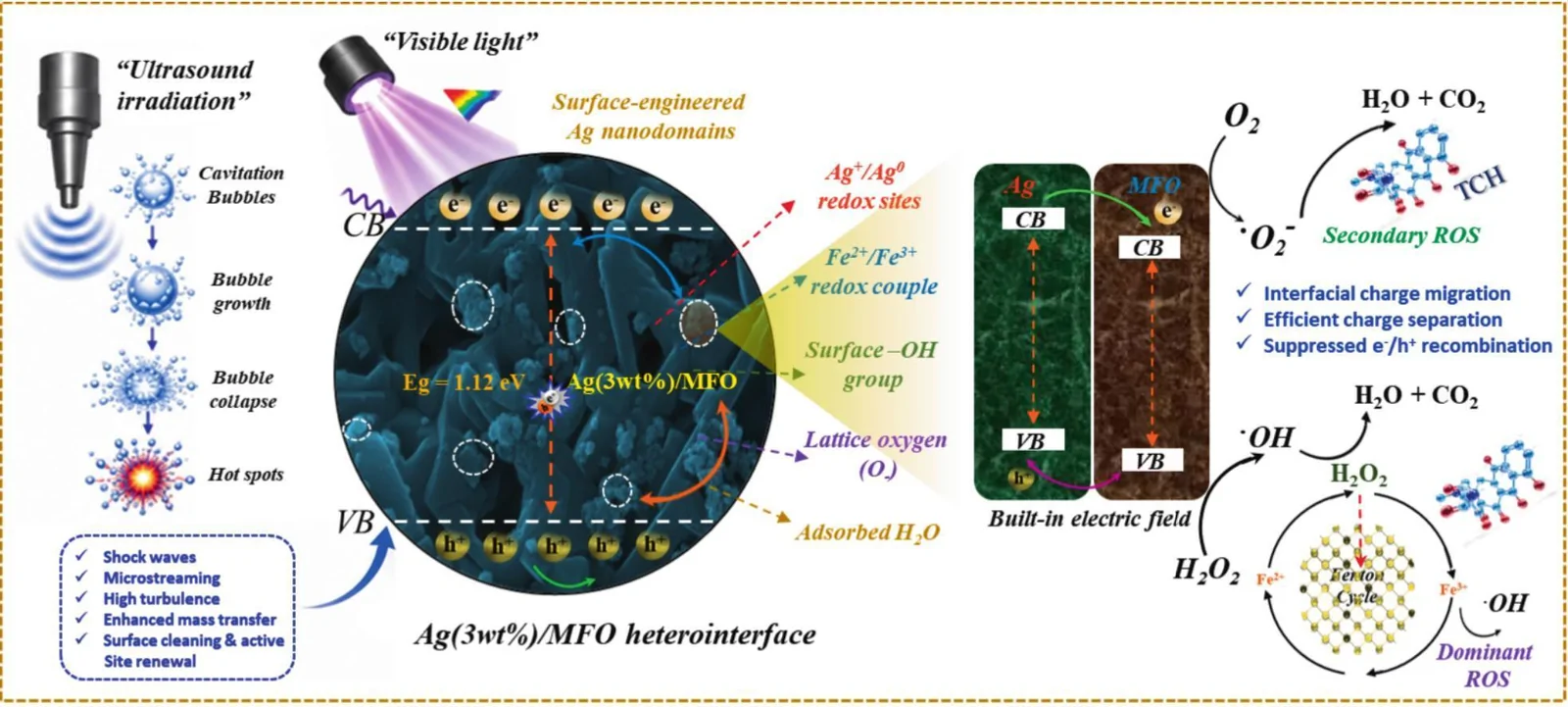

## 1 Introduction

The rapid growth of industrialization and urbanization has substantially increased freshwater consumption, resulting in the continuous discharge of large volumes of wastewater into aquatic environments [1], [2]. Among the diverse classes of emerging contaminants, pharmaceutical residues have attracted considerable attention because of their persistence, biological activity, and incomplete removal by conventional wastewater treatment technologies. Antibiotics are of particular concern due to their widespread consumption in human healthcare, veterinary medicine, and agriculture. According to the World Health Organization (WHO), approximately 70 % of administered antibiotics ultimately enter environmental water systems through pharmaceutical manufacturing, agricultural runoff, and municipal wastewater discharges [3], [4], [5]. Tetracycline (TCH), one of the most extensively used broad-spectrum antibiotics, is frequently detected in surface water, groundwater, and wastewater owing to its chemical stability, strong affinity for environmental matrices, and resistance to biodegradation [6], [7]. Its continuous release not only threatens aquatic ecosystems but also accelerates the dissemination of antibiotic-resistant microorganisms, creating a significant environmental challenge. Consequently, developing efficient and sustainable technologies for antibiotic removal remains an important research priority.

Advanced oxidation processes (AOPs) have emerged as one of the most effective approaches for eliminating refractory organic contaminants through the generation of highly reactive oxygen species (ROS), particularly hydroxyl (•OH) and superoxide (•O_2_^−^) radicals [8]. Among them, visible-light (VL)-driven photocatalytic systems have received extensive attention because they utilize solar energy while operating under relatively mild conditions. Nevertheless, many conventional semiconductor photocatalysts, including TiO_2_, ZnO, CuO, Fe_2_O_3_, and SnO_2_, still suffer from rapid electron-hole recombination, limited VL utilization, and insufficient long-term stability, restricting their practical applications [9]. Integrating ultrasonic irradiation with photocatalysis provides an effective strategy to overcome these limitations. Acoustic cavitation enhances mass transfer, continuously refreshes catalyst surfaces, accelerates H_2_O_2_ activation, and promotes ROS generation, thereby significantly improving overall photo-Fenton efficiency.

Spinel ferrites have recently emerged as attractive photo-Fenton catalysts because of their narrow band gaps, magnetic recoverability, low cost, and structural stability [10]. Among them, magnesium ferrite (MgFe_2_O_4_, MFO) has attracted increasing interest owing to its VL response, soft magnetic behavior, and environmentally benign composition [11]. However, the photocatalytic efficiency of pristine MFO is still constrained by sluggish charge transport and rapid recombination of photogenerated charge carriers. Surface engineering represents an effective strategy to address these challenges by constructing heterointerfaces that facilitate charge separation and improve interfacial electron migration. In particular, noble metal Ag has attracted considerable interest because of its excellent electrical conductivity, electron-trapping capability, and ability to promote H_2_O_2_ activation, making it highly effective for enhancing photo-Fenton reactions [12]. Recent studies on Ag-modified ferrite-based photocatalysts have reported improved photocatalytic activity through enhanced VL adsorption and charge separation [13]. However, most reported Ag/ferrite systems have been investigated under conventional photocatalytic or photo-Fenton conditions. In contrast, the use of an Ag/MgFe_2_O_4_ heterointerface in an US/VL-photo-Fenton system for TCH degradation has received limited attention. Therefore, the present study focuses on Ag(3 wt%)/MFO as a surface-engineered catalyst for integrating the advantages of Ag modification, ferrite-based photo-Fenton activity, and ultrasonic assistance.

In this study, pristine MFO together with surface-engineered Zr(3 wt%)/MFO, Co(3 wt%)/MFO, and Ag(3 wt%)/MFO photocatalysts were synthesized through a dual-ion co-precipitation route followed by wet impregnation. The US/VL-photo-Fenton performances were systematically evaluated for TCH degradation. Among the prepared photocatalysts, Ag(3 wt%)/MFO exhibited the highest activity owing to the formation of efficient Ag/MFO heterointerfaces that promoted interfacial charge transfer, accelerated Fe^2+^/Fe^3+^ redox cycling, and enhanced ROS generation. The catalyst demonstrated excellent structural stability and recyclability during repeated operation. Furthermore, radical quenching experiments, LC-MS analysis, and density functional theory (DFT) calculations were employed to elucidate the reaction mechanism, degradation pathway, and molecular-level reactive sites responsible for TCH oxidation. These findings provide valuable insights into the rational design of efficient ultrasound-assisted ferrite-based photo-Fenton catalysts for sustainable wastewater remediation.

## 2 Experimental details

### 2.1 Materials and reagents

Magnesium nitrate hexahydrate (Mg (NO_3_)_2_·6H_2_O) and ferric nitrate nonahydrate (Fe (NO_3_)_3_·9H_2_O) were employed as the magnesium and iron precursors for the synthesis of MFO. Citric acid served as the complexing agent during the preparation of the ferrite precursor. Silver nitrate (AgNO_3_), cobalt (II) nitrate hexahydrate (Co (NO_3_)_2_ ·6H_2_O), and zirconium oxychloride hexahydrate (ZrOCl_2_·6H_2_O) were selected as surface-modifying metal precursors, while ethanol was used as the dispersion medium during the wet impregnation process. Hydrogen peroxide (H_2_O_2_, 30% w/w) was employed as the oxidant in the US/VL-photo-Fenton experiments to facilitate ROS generation. The initial solution pH was adjusted using hydrochloric acid (HCl) and sodium hydroxide (NaOH). TCH was chosen as the model antibiotic pollutant to evaluate the photocatalytic performance of the prepared materials. Radical scavenging experiments were performed using isopropyl alcohol (IPA), benzoquinone (BQ), and AgNO_3_ as selective scavengers for hydroxyl radicals (•OH), superoxide radicals (•O_2_^−^), and photogenerated electrons (e^-^), respectively. All chemicals were of analytical grade and were used as received without further purification.

### 2.2 Preparation of MFO photocatalyst

The MFO photocatalyst was synthesized using a modified co-precipitation method based on the procedure reported by Kim et al. [14]. Initially, 0.1 M Mg(NO_3_)_2_·6H_2_O and 0.2 M Fe(NO_3_)_3_·9H_2_O were dissolved in 0.1 M citric acid, maintaining a Mg and Fe molar ratio of 1:2. The precursor solution was magnetically stirred for 20 min to obtain a homogeneous mixture. Subsequently, NaOH solution was added dropwise until the pH reached 13, inducing the precipitation of the ferrite precursor. The resulting suspension was maintained at 80 °C under continuous stirring for an additional 20 min to complete the precipitation process. The brown precipitate was separated by centrifugation at 8000 rpm for 10 min, thoroughly washed with deionized water followed by ethanol to remove residual impurities, and then dried at 70 °C for 24 h. The dried precursor was calcined in a muffle furnace at 800 °C for 5 h at a heating rate of 5 °C min^-1^. Finally, the calcined product was ground into a fine powder and used for the subsequent surface engineering and photocatalytic experiments.

### 2.3 Fabrication of Surface-Engineered MFO photocatalysts

**Scheme 1:**
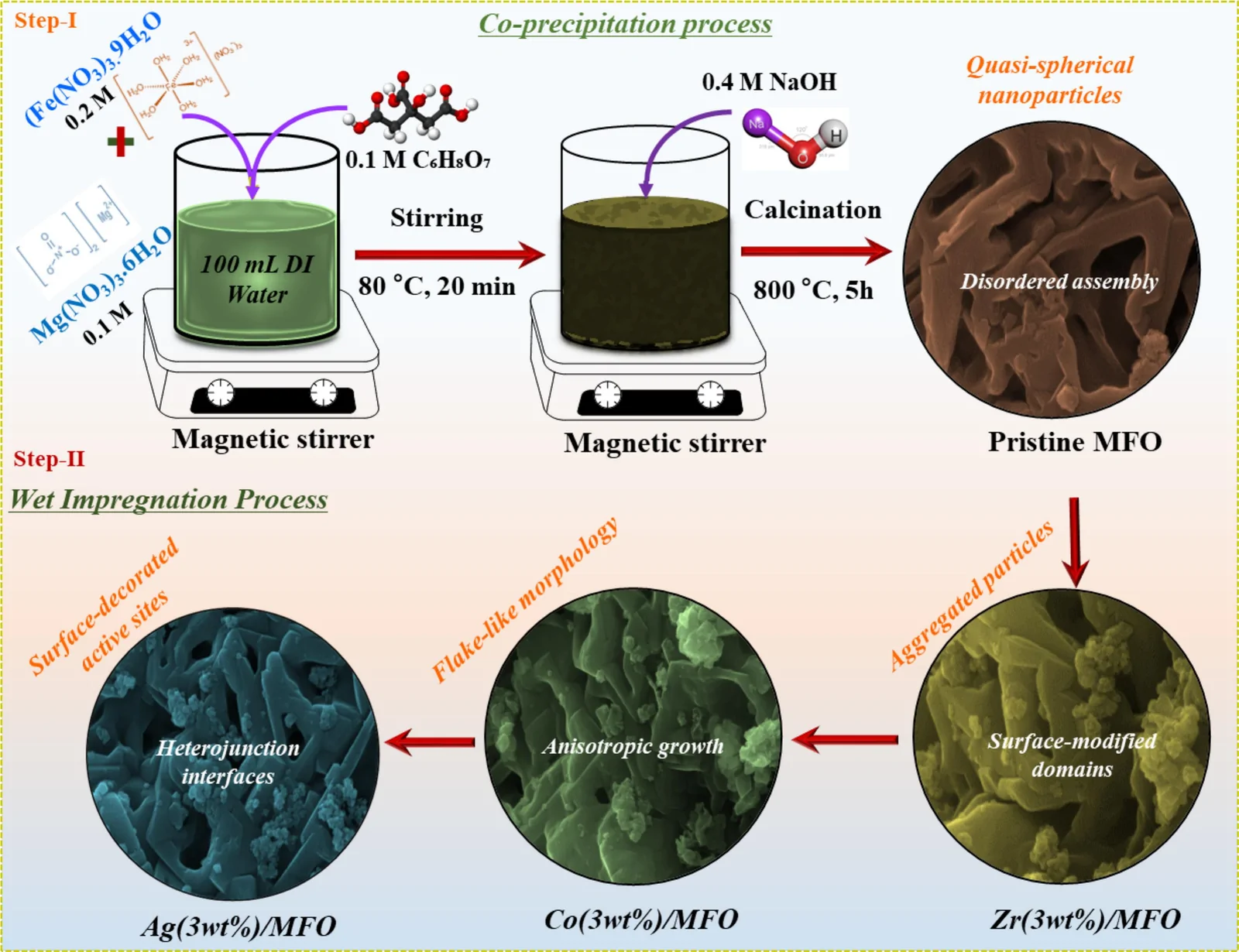
Schematic illustration of the co-precipitation-assisted synthesis of pristine MFO followed by surface engineering via wet impregnation to obtain Zr(3 wt%)/MFO, Co(3 wt%)/MFO, and Ag(3 wt%)/MFO photocatalysts.

Surface-engineered MFO photocatalysts were prepared using a wet impregnation method adapted from the procedure reported by Hwang et al. [15]. Briefly, 110 mg of the as-prepared MFO powder was mixed with the calculated amount of Zr, Co, and Ag precursors to obtain 3 wt% metal-loaded composites. The mixture was thoroughly ground with a small volume of ethanol in an agate mortar to ensure homogeneous dispersion of the metal species over the MFO surface. The resulting powders were then calcined at 300 °C for 2 h, allowing the deposited species to firmly anchor onto the MFO surface while promoting the formation of stable heterointerfaces and minimizing particle agglomeration. The synthesized photocatalysts were designated as Zr(3wt%)/MFO, Co(3wt%)/MFO, and Ag(3wt%)/MFO, according to the incorporated metal species and loading amount. A schematic illustration of the synthesis procedure is presented in Scheme 1.

### 2.4 Characterization techniques

The crystal structure and phase purity of the prepared photocatalysts were examined by X-ray diffraction (XRD, Philips PW 150) using Cu Kα radiation (λ=1.5406 Å). Diffraction patterns were recorded over a 2θ range of 10-80° with a step of 0.02°. Functional groups were analyzed using Fourier- transform infrared spectroscopy (FT-IR, PerkinElmer Spectrum 100) within the wavenumber range of 4000-400 cm^-1^. Surface morphology and elemental distribution were characterized by field-emission scanning electron microscopy (FE-SEM, Hitachi S-4800) equipped with energy-dispersive X-ray spectroscopy (EDS). The surface elemental composition and chemical states were investigated using X-ray photoelectron spectroscopy (XPS, PHI VersaProbe III), with all binding energies referenced to the C 1s peak at 248.8 eV. High-resolution spectra were collected using a pass energy of 23.5 eV, an energy step of 0.1 eV, and averaged over five scans. The textural properties of the photocatalysts were evaluated by N_2_ adsorption-desorption measurements at 77 K using a Quantachrome Autosorb iQ analyzer. The specific surface area and pore size distribution were determined using the Brunauer-Emmett-Teller (BET) and Barrett-Joyner-Halenda (BJH) methods, respectively. Photoluminescence (PL) spectra were recorded with an excitation wavelength of 370 nm using an Edinburgh Analytical FE30 Fluorescence Spectrometer. The degradation intermediates of TCH were identified by liquid chromatography-tandem mass spectrometry (LC-MS, Waters Xevo TQ-S micro) operated in positive electrospray ionization mode and an m/z range of 50-500.

### 2.5 US/VL-Photo-Fenton Degradation of TCH

The photocatalytic performance of pristine MFO, Zr(3wt%)/MFO, Co(3wt%)/MFO, and Ag(3wt%)/MFO was evaluated for the degradation of TCH under US/VL-photo-Fenton conditions. In a typical experiment, the desired amount of photocatalyst (20, 30, or 40 mg) was dispersed in 100 mL of TCH solution with initial concentrations of 10, 20, or 30 mg L-1. The suspension was magnetically stirred in the dark for 30 min to establish adsorption-desorption equilibrium. Subsequently, predetermined volumes of 30 wt% H_2_O_2_ were added, and the initial solution pH was adjusted to 3.0, 4.0, 5.8, 7.0, and 9.0 using 0.1 M HCl or 0.1 M NaOH. The reaction was carried out in a 150 mL quartz reactor immersed in an ultrasonic bath (JPL Sonic 3MX, China) operated at an output power of 120 W and a fixed frequency of 37 kHz. Simultaneously, the reactor was irradiated with a 300 W Xe lamp equipped with a λ ≥ 420 nm cutoff filter positioned 15 cm above the reaction vessel, providing an incident light intensity of approximately 120 mW cm^-2^. Continuous magnetic stirring was maintained throughout the reaction to ensure uniform suspension of the photocatalyst. The US/VL-photo-Fenton process was performed for 120 min, and 1 mL aliquots were withdrawn at 20 min intervals. The collected samples were immediately centrifuged at 5000 rpm for 10 min, and the supernatant was analyzed using a UV-Vis spectrophotometer to determine the residual TCH concentration. The degradation efficiency was calculated using Eq. (1).

(1) $$\text{Degradation percentage}\;{=}_{\;}\frac{C_{0}-C_{t}}{C_{0}}\times 100$$

Where C_0_ and C_t_ represent the initial concentration and the concentration at reaction time t, respectively.

### 2.6 Reusability Test

The reusability of the Ag(3wt%)/MFO photocatalyst was evaluated through four consecutive US/VL-photo-Fenton degradation cycles under the optimized reaction conditions. After each cycle, the photocatalyst was recovered by filtration using Whatman No. 42 filter paper, thoroughly washed with ethanol to remove residual organic species, and dried at 80 °C before reuse. The regenerated photocatalyst was subsequently dispersed in a fresh 100 mL TCH solution for the next degradation cycle. The photocatalytic performance was determined after each cycle to evaluate the catalyst stability and recyclability. In addition, the structural integrity of the recovered photocatalyst was further examined by post-reaction XRD and XPS analyses.

## 3 Results and discussion

### 3.1 Crystal Structure, Surface Chemistry, and Optical Characterization of MOF-based Photocatalysts

**Fig. 1:**
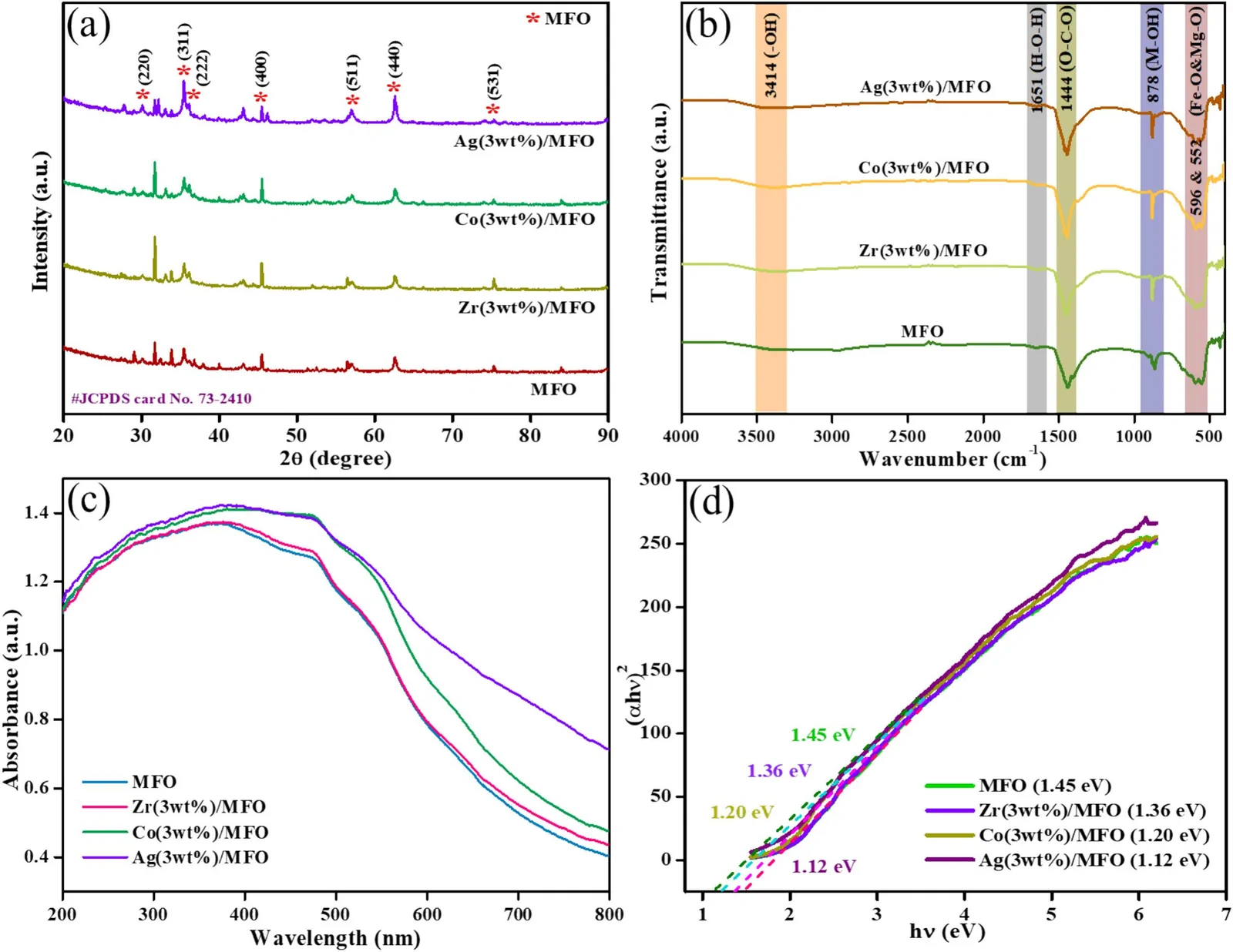
Structural and optical properties of pristine MFO and surface-engineered MFO composites modified with 3 wt% Zr, Co, and Ag: (a) XRD patterns, (b) FT-IR spectra, (c) UV–Vis DRS spectra, and (d) corresponding Tauc plots for band-gap energy estimation.

The phase composition and crystal structure of pristine MFO and surface-engineered Zr(3wt%)/MFO, Co(3wt%)/MFO, and Ag(3wt%)/MFO photocatalysts were examined by XRD, and the corresponding patterns are presented in Fig. 1a. All samples exhibited the characteristic diffraction reflections of cubic spinel MFO, which agree well with the standard diffraction data (JCPDS No. 73-2410, space group Fd-3m), confirming the successful formation of a phase-pure crystalline framework. The diffraction peaks located approximately at 2θ = 30.2^°^, 35.5^°^, 36.3^°^, 45.5^°^, 57.1^°^, 62.6^°^, and 75.4^°^ are indexed to the (220), (311), (222), (400), (511), (440), and (533) crystallographic planes, respectively. No discernible diffraction peaks corresponding to crystalline Zr, Co, and Ag phases were detected in the modified samples, suggesting that the deposited oxide species are highly dispersed on the MFO surface or remain in an amorphous state below the detection limit of XRD. Moreover, the diffraction peak positions and profiles remained essentially unchanged following surface modification, indicating that the spinel lattice was preserved without detectable lattice distortion or bulk cation substitution. These observations confirm that the introduced metal oxides are predominantly confined to the catalyst surface, where they establish interfacial contact with the MFO matrix rather than being incorporated into the crystal lattice. Similar structural characteristics have been reported for surface-modified MFO photocatalysts, where the cubic spinel phase is retained after the incorporation of secondary oxide species without the formation of crystalline impurity phases [16], [17]. Overall, the XRD results demonstrate that the applied surface engineering strategy preserves the structural integrity of MFO while introducing surface-localize oxide species. The retained crystalline framework provides a stable structural basis for the subsequent US/VL-photo Fenton process.

The FT-IR spectra of pristine MFO and surface-modified Zr(3wt%)/MFO, Co(3wt%)/MFO, and Ag(3wt%)/MFO are presented in Fig. 1b. The spectra reveal that surface modification does not alter the fundamental chemical framework of MOF, while introducing subtle changes in the local bonding environment. A broad absorption band centered at approximately 3414 cm^1^ is assigned to the stretching vibration of hydroxyl groups and physisorbed water molecules, whereas the band at 1651 cm^−1^ corresponds to the H-O-H binding vibration of absorbed water [18]. These oxygen-containing surface species are expected to provide accessible active sites for ROS generation during the US/VL-photo Fenton process. Weak adsorption bands observed at approximately 1444 cm^−1^ and 878 cm^−1^ are attributed to carbonate-related O-C-O vibrations and M-OH-associated surface species, respectively, originating from atmospheric CO_2_ adsorption and surface hydroxylation [19]. Their presence indicates a hydroxyl-rich surface environment without affecting the bulk crystal structure. The characteristic metal-oxygen vibrations of the spinel ferrite lattice appear at 596 cm^−1^ and 552 cm^−1^, corresponding to Fe-O stretching at octahedral sites and Mg-O vibrations at tetrahedral sites, respectively. The retention of these bands across all samples confirms that the spinel framework remains intact following oxide deposition. No additional vibrational features attributable to Zr, Co, and Ag were detected, suggesting that these species are highly dispersed over the MFO surface or possess insufficient crystallinity for FT-IR detection. Minor variations in band intensity after modification indicate interfacial interactions between the deposited oxides and the MFO matrix, consistent with previous reports on surface-engineered MFO photocatalysts [20]. Overall, the FT-IR demonstrates that the surface engineering strategy preserves the intrinsic bonding network of MFO while increasing the abundance of hydroxylated surface sites. These characteristics are expected to favor interfacial redox reactions during the subsequent US/VL-photo Fenton process without compromising the structural integrity of the catalyst.

The optical properties of pristine MFO and surface-engineered Zr(3wt%)/MFO, Co(3wt%)/MFO, and Ag(3wt%)/MFO were investigated by UV-Vis DRS (Fig. 1c). All photocatalysts exhibited the ability to utilize VL irradiation. Following surface modification, the absorption edge gradually shifted toward longer wavelengths, reflecting changes in the electronic structure induced by interfacial coupling between MFO and the deposited oxide species. The corresponding Tauc plots (Fig. 1d) revealed a progressive reduction in the optical band-gap energy from 1.45 eV for pristine MFO to 1.36, 1.20 eV, and 1.12 eV for Zr(3wt%)/MFO, Co(3wt%)/MFO, and Ag(3wt%)/MFO, respectively. This trend indicates that surface oxide deposition modifies the electronic environment of MFO, creating favorable energy states that facilitate VL absorption. Among the investigated photocatalysts, Ag(3wt%)/MFO exhibited the smallest band gap and subsequently demonstrated the highest catalytic efficiency. Nevertheless, optical absorption alone cannot account for the observed activity enhancement. In heterogeneous catalytic systems, overall performance is governed by the combined influence of light harvesting, interfacial charge migration, defect-state distribution, and surface reaction kinetics [21]. The superior behavior of Ag(3wt%)/MFO is therefore attributed to the synergistic modulation of its optical and electronic properties, which promotes more efficient utilization of photogenerated charge carriers while suppressing electron-hole recombination. Overall, the UV-Vis DRS results demonstrate that surface engineering effectively tailors the electronic structure of MFO without disrupting its crystalline framework. The optimized optical response is expected to improve photon utilization and support efficient interfacial redox reactions during the US/VL-photo Fenton process.

### 3.2 Morphological and Elemental Distribution Analysis of MFO-Based Photocatalysts

**Fig. 2:**
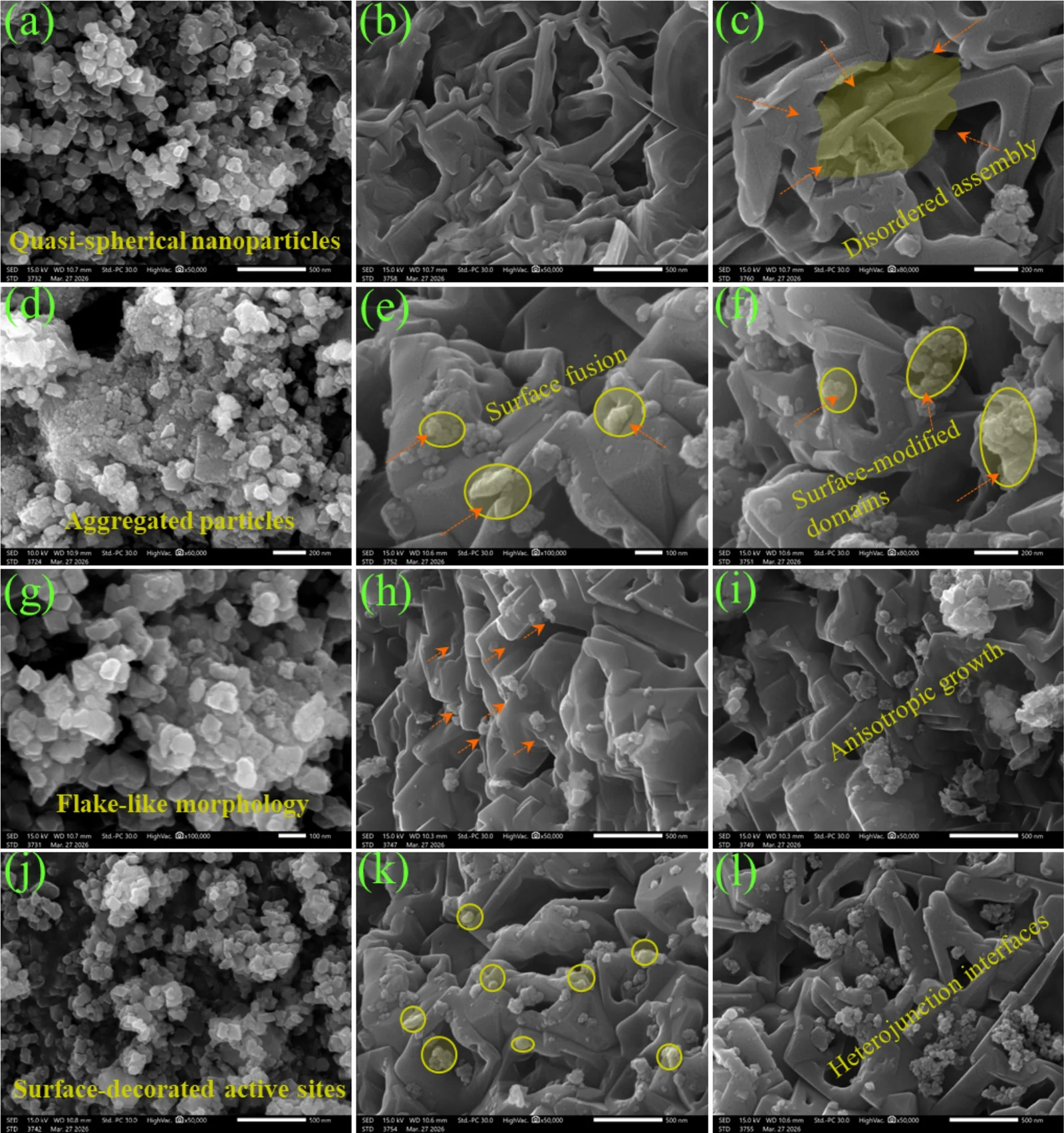
SEM images of pristine MFO (a-c), Zr(3 wt%)/MFO (d-f), Co(3 wt%)/MFO (g-i), and Ag(3 wt%)/MFO (j-l).

The morphological characteristics of pristine MFO and surface-engineered Zr(3wt%)/MFO, Co(3wt%)/MFO, and Ag(3wt%)/MFO photocatalysts were examined by FESEM, and the corresponding micrographs are presented in Fig. 2(a-l). Pristine MFO (Fig. 2a-c) consists of agglomerated quasi-spherical particles with irregular packing, resulting in a relatively compact morphology. After surface modification, distinct changes in particle texture and surface architecture were observed while the overall framework of MFO remained preserved. The Zr(3wt%)/MFO (Fig. 2d-f) sample exhibits a partially decorated particle surface with finely dispersed nanoscale domains, indicating uniform deposition of Zr over the MFO matrix. In contrast, Co(3wt%)/MFO develops a more open and interconnected morphology composed of flake-like features, producing a rougher surface texture than pristine MFO (Fig. 2g-i). The Ag(3wt%)/MFO (Fig. 2j-l) photocatalyst displays uniformly distributed nanoscale domains with minimal localized aggregation, suggesting an intimate interfacial connection between Ag and the MFO substrate. The homogeneous dispersion of Ag indicates effective surface engineering without disrupting the original particle morphology. The observed morphological evolution demonstrates that the deposited oxide species surface growth behavior occurs through interfacial interactions rather than bulk structural reconstruction. Similar morphology modulations have been reported for oxide-modified spinel ferrite photocatalysts, where surface engineering promotes the formation of well-dispersed heterointerfaces while preserving the parent architecture [22]. For the present system, the uniformly distributed Ag nanodomains are expected to provide abundant catalyst solution contact sites and maintain stable interfacial regions during the US/VL-photo Fenton process. Such morphological characteristics are favorable for efficient mass transport under cavitation while preserving the structural integrity of the photocatalyst throughout repeated cycles [23].

To further verify the elemental composition and spatial distribution of the deposited species, FESEM-coupled EDS analysis together with elemental mapping was carried out for the pristine MFO and surface-engineered Zr(3wt%)/MFO, Co(3wt%)/MFO, and Ag(3wt%)/MFO photocatalysts. The corresponding EDS spectra, quantitative elemental compositions, and mapping are presented in Fig. S1(a-l). Pristine MFO exhibited only O, Fe, and Mg signals with elemental weight percentages of 61.09 wt% O, 24.36 wt% Fe, and 14.55 wt% Mg, in agreement with the expected stoichiometry of the spinel ferrite. The elemental maps further reveal a homogeneous distribution of O, Fe, and Mg throughout the analyzed region, indicating compositional uniformity without detectable impurities. Following surface engineering, characteristic signals of Zr, Co, and Ag emerged in the respective composites while maintaining the Mg, Fe, and O framework. The Zr(3wt%)/MFO photocatalyst contained 64.01 wt% O, 22.41 wt% Fe, 10.05 wt% Mg, and 3.53 wt% Zr, wheras Co(3wt%)/MFO exhibited 66.75 wt% O, 19.76 wt% Fe, 10.43 wt% Mg, and 3.06 wt% Co. Likewise, Ag(3wt%)/MFO consisted of 65.15 wt% O, 20.60 wt% Fe, 11.15 wt% Mg, and 3.10 wt% Ag. The measured compositions closely correspond to the designed 3 wt% oxide loading, demonstrating the effectiveness and reproducibility of the surface modification strategy. Elemental mapping further shows that Zr, Co, and Ag are evenly distributed throughout the MFO matrix without detectable segregation or isolated secondary domains. These observations are consistent with the XRD results, which revealed no crystalline impurity phases, indicating that the introduced oxide species are predominantly confined to the catalyst interface rather than incorporated into the bulk lattice. Such compositional homogeneity is expected to preserve stable interfacial contact between MFO and the deposited oxides during the US/VL-photo Fenton process, providing a robust platform for subsequent interfacial redox reactions [24].

### 3.3 TEM Investigation of Ag(3wt%)/MFO Photocatalyst

**Fig. 3:**
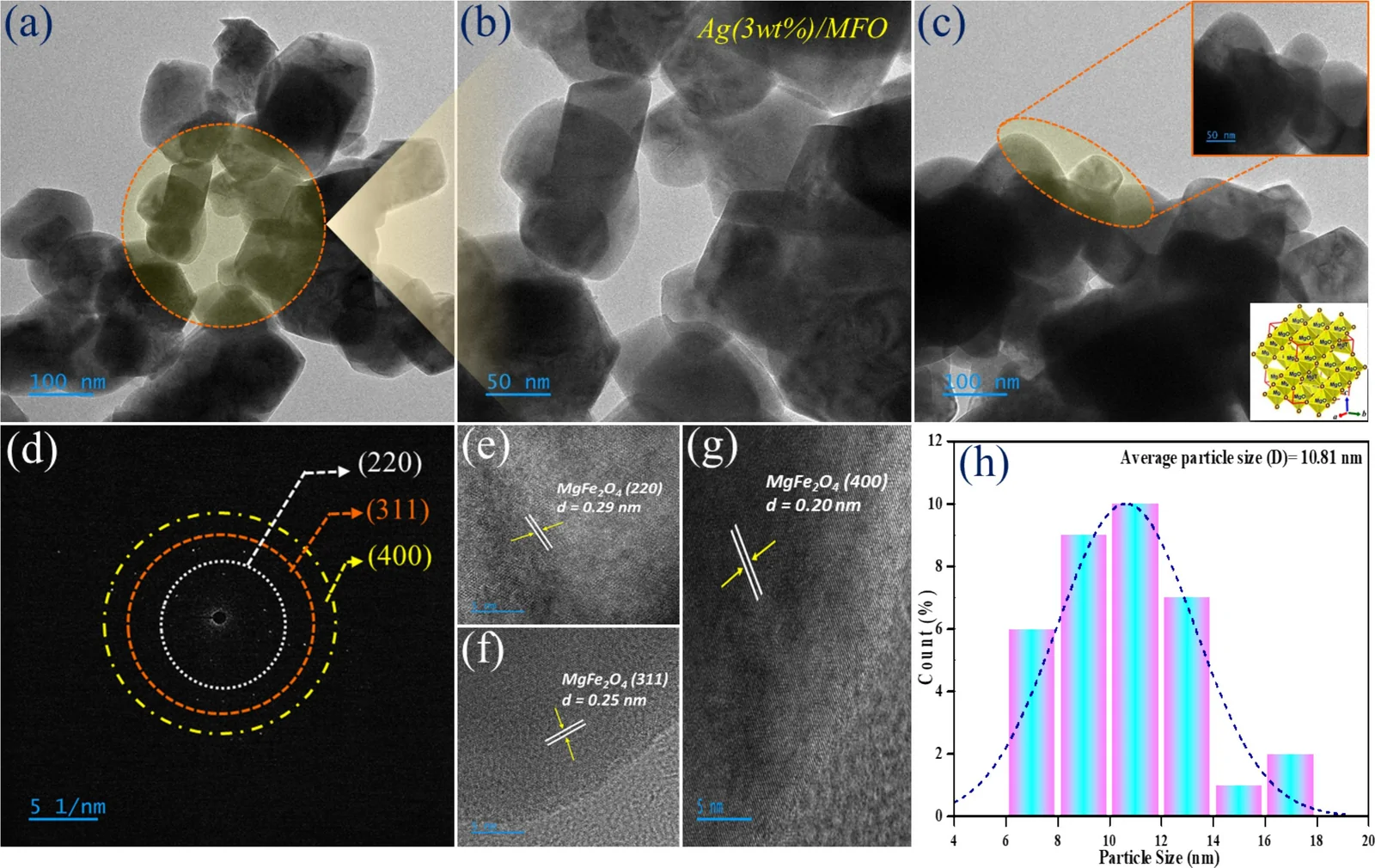
TEM images (a–c), SAED pattern (d), HR-TEM images with lattice fringes (e-g), and particle size distribution (h) of Ag(3 wt%)/MFO photocatalyst.

The microstructure of the optimized Ag(3wt%)/MFO photocatalyst was further examined by TEM, SAED, and HR-TEM, and the corresponding images are presented in Fig. 3(a–h). The TEM micrographs (Fig. 3a–c) reveal uniformly distributed nanoparticles with well-preserved morphology, where finely dispersed Ag nanodomains are anchored onto the MFO matrix without forming large aggregates. The intimate contact between the deposited oxide and the ferrite support indicates the successful construction of a surface-engineered heterostructure. Higher-magnification images further demonstrate that the deposited Ag is distributed as nanoscale domains over the MFO surface, producing a continuous interfacial region rather than isolated clusters. Such a morphology is consistent with the controlled nucleation and surface deposition achieved through the combined co-precipitation and wet-impregnation synthesis route. The SAED pattern (Fig. 3d) exhibits well-defined concentric diffraction rings, confirming the polycrystalline nature of the composite. HR-TEM images (Fig. 3e-g) display distinct lattice fringes with interplanar spacings of approximately 0.29 nm, 0.25 nm, and 0.20 nm, corresponding to the (220), (311), and (400) planes of cubic spinel MFO, respectively. These lattice spacings agree well with the XRD result, demonstrating that the crystalline framework of MFO is retained after Ag deposition. No structural distortion or lattice discontinuity was observed at the interface, indicating that the surface engineering strategy preserves the crystallographic integrity of the parent ferrite. ImageJ analysis (Fig. 3h) revealed an average particle size of approximately 10.81 nm, with a relatively narrow distribution ranging from 6 to 18 nm. The small particle size, together with the homogeneous dispersion of Ag nanodomains, increases the available interfacial contact area between the deposited oxide and the MFO support. Similar nanoscale heterointerfaces have been reported for surface-engineered spinel ferrite photocatalysts, where uniform oxide dispersion contributes to the formation of stable composite architectures [6]. Overall, the TEM results verify the successful fabrication of a finely dispersed Ag(3wt%)/MFO heterostructure with preserved crystallinity and well-defined interfacial characteristics, providing a structurally robust platform for the subsequent US/VL-photo-Fenton process.

### 3.4 Chemical States and Textural Characterization

**Fig. 4:**
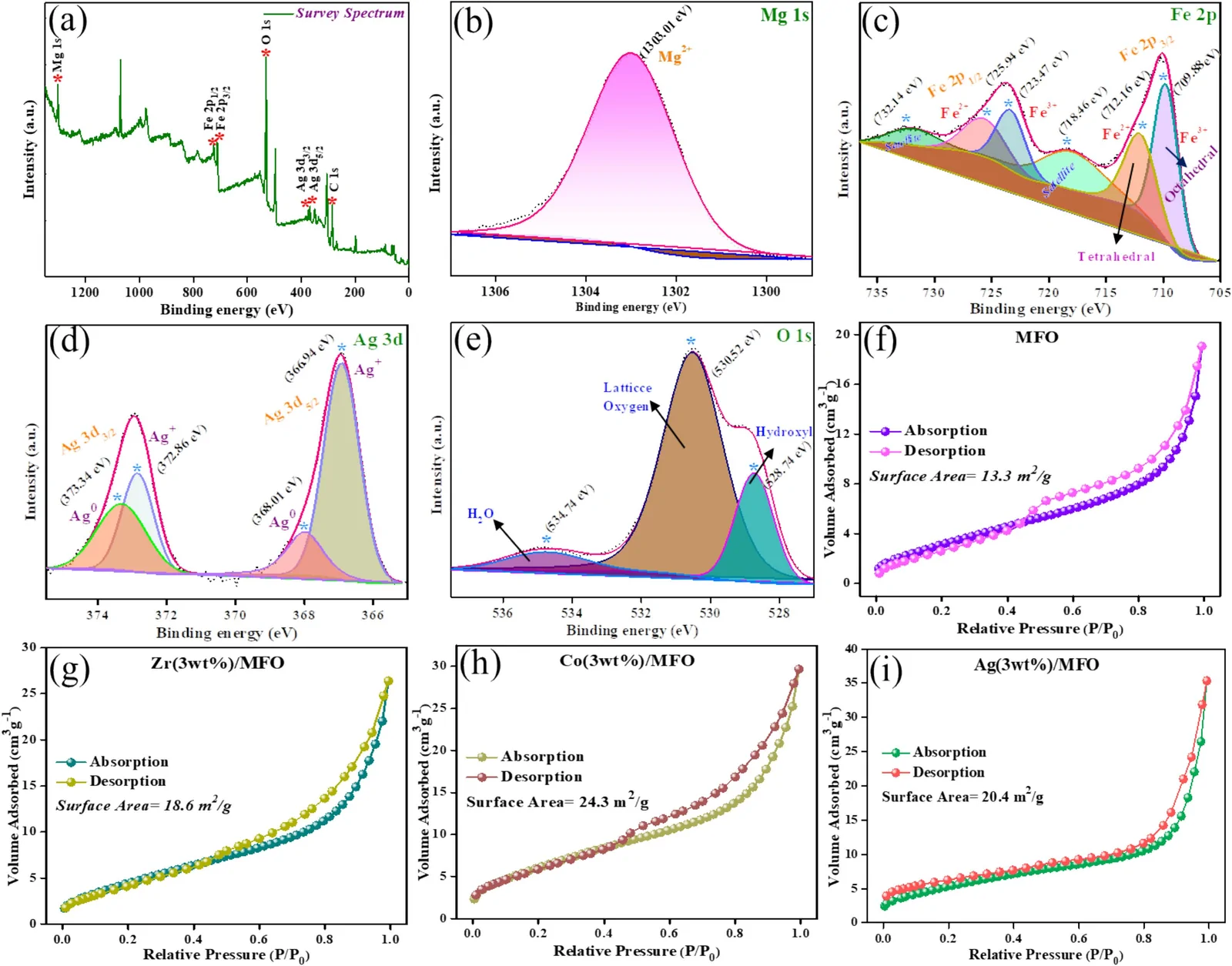
XPS characterization of the optimized Ag(3 wt%)/MFO photocatalyst: (a) survey spectrum, high-resolution spectra of (b) Mg 1 s, (c) Fe 2p, (d) Ag 3d, and (e) O 1 s. Nitrogen adsorption–desorption isotherms of (f) pristine MFO, (g) Zr(3 wt%)/MFO, (h) Co(3 wt%)/MFO, and (i) Ag(3 wt%)/MFO photocatalyst.

The surface electronic structure and chemical states of the optimized Ag(3wt%)/MFO photocatalyst were examined by XPS, and the corresponding survey and high-resolution spectra are presented in Fig. 4(a-e). The XPS analysis was employed to elucidate the surface elemental composition, oxidation states, and electronic interactions established following Ag deposition. The XPS survey spectrum (Fig. 4a) displays the characteristic core-level signals of Mg 1s, Fe 2p, Ag 3d, and O 1s, confirming the presence of all constituent elements in the near-surface region of the photocatalyst. No additional elemental signals were detected, indicating the absence of detectable surface impurities. A weak C 1s peak is also observed, which is attributed to adventitious carbon adsorbed from the atmosphere during sample handling. The high-resolution Mg 1s spectrum (Fig. 4b) exhibits a single symmetric peak centered at 1303.01 eV, characteristic of Mg^2+^ species in the spinel MFO lattice. The presence of a single Mg 1s component indicates that Ag deposition does not significantly alter the local chemical environment of magnesium. The high-resolution Fe 2p spectrum (Fig. 4c) consists of two characteristic spin-orbit components centered at 709.88 eV and 723.47 eV, corresponding to Fe 2p_3/2_ and Fe 2p_1/2_, respectively. Additional features located at 712.16 eV and 725.94 eV, together with shake-up satellite peaks at 718.46 eV and 732.14 eV, are characteristic of spinel ferrites. The overall spectral profile indicates the coexistence of Fe^2+^ and Fe^3+^ species, consistent with the mixed-valence nature of MFO reported previously [13]. Such mixed oxidation states provide redox-active centers that are expected to support continuous Fe^2+^/Fe^3+^ cycling during the US/VL-photo-Fenton process. The high-resolution Ag 3d spectrum (Fig. 4d) was deconvoluted into two pairs of spin-orbit components, revealing the coexistence of oxidized and metallic silver species. The peaks centered at 366.94 eV and 372.86 eV are assigned to Ag^+^ (Ag 3d_5/2_ and Ag 3d_3/2_), confirming the presence of Ag on the MFO surface. A second pair of peaks located at 368.01 eV and 373.34 eV corresponds to Ag^0^, indicating partial reduction of Ag^+^ during synthesis or subsequent interfacial interactions. The coexistence of Ag^+^ and Ag^0^ suggests the formation of an electronically coupled Ag/MFO interface that may facilitate interfacial electron migration under US/VL irradiation. The high-resolution O 1s spectrum (Fig. 4e) was deconvoluted into three components centered at 528.74, 530.52, and 534.74 eV. The low-binding-energy peak component is assigned to surface hydroxyl species, the dominant peak at 530.52 eV corresponds to lattice oxygen, and the high-binding-energy component is attributed to adsorbed H_2_O molecules. The coexistence of these oxygen species reflects the chemically heterogeneous surface environment established through Ag deposition. Overall, the XPS results confirm that Ag surface modification tailors the surface electronic structure of MFO while preserving its characteristic chemical states, providing direct evidence for the formation of a well-defined Ag(3wt%)/MFO heterointerface [25].

Furthermore, nitrogen adsorption-desorption analysis was performed to evaluate the textural characteristics of pristine MFO and surface-engineered Zr(3wt%)/MFO, Co(3wt%)/MFO, and Ag(3wt%)/MFO photocatalysts. The corresponding BET surface area and BJH pore-size distribution curves are presented in Fig. 4(f-i) and Fig. S2(a-d), respectively. Pristine MFO exhibits a specific surface area of 13.3 m^2^/g with an average diameter of 125.1 Å, indicating a relatively limited accessible surface area. Following surface modification, noticeable changes in the textural properties were observed. The surface area increased to 18.6 m^2^/g for Zr(3wt%)/MFO, accompanied by a reduction in the average pore diameter to 77.8 Å. The Co(3wt%)/MFO sample displayed the highest BET surface area (24.3 m^2^/g) with an average pore diameter of 71.3 Å, whereas Ag(3wt%)/MFO exhibited a surface area of 20.4 m^2^/g and an average pore diameter of 74.7 Å. These results indicate that deposition of secondary oxide species modifies the surface texture of MFO, leading to increased surface accessibility while maintaining a mesoporous framework. Surface structural features are beneficial for facilitating reactant adsorption and molecular diffusion during catalytic reactions [26]. Although Co(3wt%)/MFO possesses the largest specific surface area, its photocatalytic performance is lower than that of Ag(3wt%)/MFO, demonstrating that catalytic activity is not exclusively determined by surface area. Instead, the superior performance of Ag(3wt%)/MFO is attributed to the combined influence of its optimized interfacial electronic structure, efficient charge-transfer characteristics, and favorable surface chemistry, as evidenced by the complementary structural and spectroscopic analyses.

### 3.5 Photoluminescence Analysis

The photoluminescence spectra of pristine MFO and surface-engineered Zr(3wt%)/MFO, Co(3wt%)/MFO, and Ag(3wt%)/MFO photocatalysts are presented in Fig. S3. All samples exhibit a broad emission band centered at approximately 520 nm, which is attributed to the radiative recombination of photogenerated charge carriers. The emission intensity varies after surface modification, indicating that incorporation of Zr, Co, and Ag alters the electronic structure and recombination characteristics of the MFO matrix. Compared with pristine MFO, the modified MFO showed progressively higher emission intensities, with Ag(3wt%)/MFO exhibiting the strongest PL response. The variation suggests that surface oxide deposition modifies the available electronic states and radiative transition pathways through interfacial interactions. However, steady-state PL primarily reflects radiative recombination and does not independently determine photocatalytic performance. In heterogeneous photocatalytic systems, catalytic efficiency is governed by the combined effects of interfacial charge transfer, surface reaction kinetics, active-site availability, and reactive oxygen species generation [27]. Therefore, the superior US/VL-photo-Fenton activity of Ag(3wt%)/MFO is attributed to the synergistic contribution of its optimized heterointerface and surface electronic structure rather than the PL response alone.

### 3.6 US/VL-Photo-Fenton Degradation of TCH

**Fig. 5:**
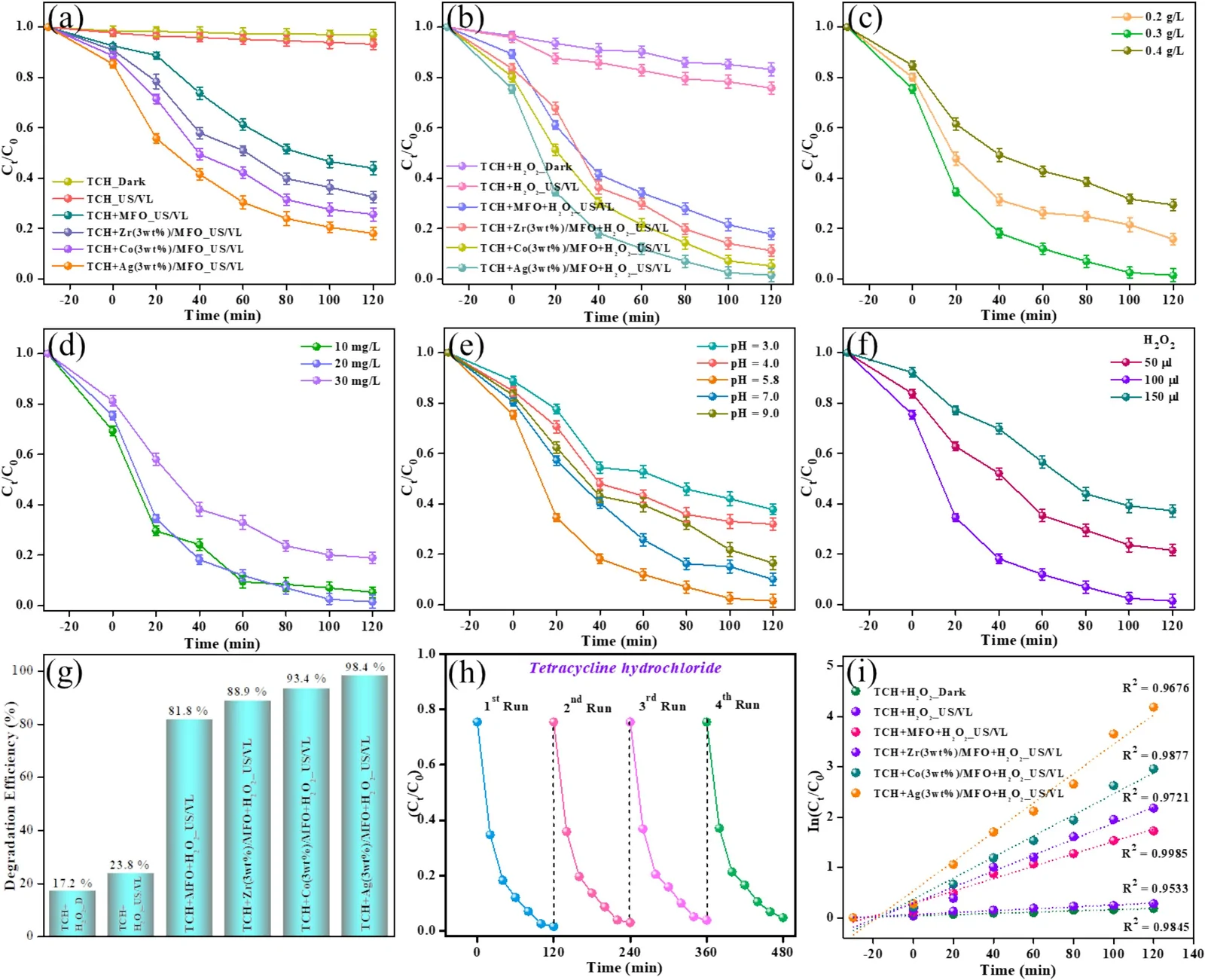
US/VL-photo-Fenton degradation performance of TCH over pristine MFO and surface-engineered Zr(3 wt%)/MFO, Co(3 wt%)/MFO, and Ag(3 wt%)/MFO photocatalysts: (a) comparison of photocatalytic degradation efficiencies, (b) contribution of different reaction components, (c) effect of catalyst dosage, (d) effect of initial TCH concentration, (e) effect of solution pH, (f) effect of H_2_O_2_ dosage, (g) comparison of different reaction systems, (h) reusability over four consecutive cycles, and (i) pseudo-first-order kinetic analysis.

The US/VL-photo-Fenton performance of pristine MFO and surface-engineered Zr(3wt%)/MFO, Co(3wt%)/MFO, and Ag(3wt%)/MFO photocatalysts was evaluated using TCH as a model antibiotic pollutant, and the corresponding results are presented in Fig. 5(a-i). As shown in Fig. 5a, negligible TCH removal was observed in the absence of the catalyst, with degradation efficiencies of only 3% under dark conditions and 6.4% under combined US/VL irradiation, confirming that direct sonolysis and photolysis contribute minimally to pollutant decomposition. In contrast, the pristine MFO achieved 55.7% degradation within 120 min, demonstrating its inherent catalytic activity under US/VL-photo-Fenton conditions. Surface engineering progressively enhanced the degradation efficiency from 67.8% for Zr(3wt%)/MFO, 76.4% for Co(3wt%)/MFO, and finally 82% for Ag(3wt%)/MFO. Among the investigated photocatalysts, Ag(3wt%)/MFO exhibited the highest activity, suggesting that Ag surface engineering establishes the most effective heterointerface for the US/VL-photo-Fenton process. To further clarify the contribution of each reaction component, a series of control experiments was performed (Fig. 5b). The complete system comprising Ag(3wt%)/MFO, H_2_O_2_, US, and VL irradiation exhibited the highest degradation efficiency (98.4%), whereas all reference systems showed substantially lower activity. In the absence of the photocatalyst, only 17.2% TCH degradation was achieved using H_2_O_2_ under dark conditions, while the combination of H_2_O_2_, US, and VL irradiation increased the degradation efficiency slightly to 23.8%, indicating that neither direct oxidation nor the combined US and VL alone is sufficient for efficient TCH removal. Upon introducing the photocatalyst, the degradation efficiencies increased markedly to 81.8%, 88.9%, and 93.4% for pristine MFO, Zr(3wt%)/MFO, and Co(3wt%)/MFO, respectively, before reaching 98.4% for Ag(3wt%)/MFO. The superior activity of Ag(3wt%)/MFO demonstrates the synergistic interaction among US irradiation, VL excitation, H_2_O_2_ activation, and the engineered Ag/MFO heterointerface. Under ultrasonic irradiation, acoustic cavitation continuously generates localized hot spots, shock waves, and microstreaming that enhance mass transfer and continuously renew the catalyst surface. Simultaneously, VL irradiation activates the Ag/MFO heterostructure, while H_2_O_2_ serves as both an oxidant and electron acceptor, promoting the generation of ROS responsible for TCH degradation [28], [29].

Subsequently, the operational parameters influencing the US/VL-photo-Fenton degradation of TCH were systematically optimized using the Ag(3wt%)/MFO photocatalyst. As shown in Fig. 5c, the catalyst dosage significantly affected the degradation efficiency. Increasing the catalyst loading from 0.2 g/L to 0.3 g/L improved the TCH removal efficiency from 84.1% to 98.4%, owing to the increased availability of catalytically active sites and enhanced activation of H_2_O_2_. However, a further increase to 0.4 g/L reduced the degradation efficiency to 70.2%. Excessive catalyst loading increases suspension turbidity, limiting VL penetration and reducing the effective utilization of ultrasonic energy. Moreover, particle agglomeration decreases the exposed active surface area and restricts mass transfer between TCH molecules and reactive species, thereby suppressing ROS generation [6]. Therefore, 0.3 g/L was selected as the optimum catalyst dosage for subsequent experiments. The influence of the initial TCH concentrations was subsequently investigated using 10, 20, and 30 mg/L solutions (Fig. 5d). Among the tested concentrations, 20 mg/L exhibited the highest degradation efficiency, indicating an appropriate balance between pollutant concentration and reactive oxygen species availability. At 10 mg/L, although ROS generation remained sufficient, the relatively low number of TCH molecules reduced the probability of effective oxidation. Conversely, increasing the concentration to 30 mg/L resulted in lower degradation efficiency because excess pollutant molecules competed for the limited active sites and reactive oxygen species. In addition, the higher solution opacity reduced VL penetration, thereby weakening photocatalytic activation and ROS production. The effect of the initial solution pH was further evaluated over the pH range of 3.0-9.0 (Fig. 5e). The maximum degradation efficiency was obtained at pH 5.8, which closely corresponds to the point of zero charge (pHpzc) of the MFO photocatalyst. Under this condition, favorable interfacial interactions between the catalyst surface and TCH molecules promote efficient adsorption, electron transfer, and H_2_O_2_ activation. Moving toward either acidic or alkaline conditions reduced the degradation efficiency because unfavorable surface charge characteristics weakened pollutant adsorption and limited the effective utilization of ROS [30]. The H_2_O_2_ dosage also played an important role in determining the degradation performance (Fig. 5f). Increasing the H_2_O_2_ dosage from 50 µL to 150 µL enhanced TCH degradation by supplying sufficient oxidant for ROS generation. However, a further increase to 150 µL slightly decreased the degradation efficiency because excess H_2_O_2_ consumed hydroxyl radicals through self-scavenging reactions. Thereby decreasing the concentration of reactive oxidizing species available for pollutant degradation.

After optimizing the reaction conditions, comparative degradation experiments were performed to elucidate the individual and synergistic contributions of the catalyst, H_2_O_2_, US, and VL irradiation to the overall US/VL-photo-Fenton process (Fig. 5g). In the absence of the photocatalyst, TCH+H_2_O_2_ under dark achieved only 17.2% degradation, while simultaneous US/VL irradiation slightly increased the degradation efficiency to 23.8%, confirming that direct oxidation, sonolysis, and photolysis contribute minimally to TCH removal. Upon introducing the photocatalyst, the degradation efficiencies increased markedly to 81.8%, 88.9%, 93.4%, and 98.4% for pristine MFO, Zr(3wt%)/MFO, Co(3wt%)/MFO, and Ag(3wt%)/MFO, respectively. The progressive enhancement clearly demonstrates the beneficial role of surface engineering in improving catalytic activity, with Ag(3wt%)/MFO exhibiting the highest degradation efficiency. Furthermore, the comparative results summarized in Table S1 demonstrate the competitive photocatalytic performance of the Ag(3wt%)/MFO photocatalyst compared with previously reported ferrite-based photocatalysts under similar reaction conditions. This superior performance originates from the synergistic interaction between acoustic cavitation, VL excitation, H_2_O_2_ activation, and the engineered Ag/MFO heterointerface. Ultrasonic irradiation continuously generates cavitation bubbles whose collapse produces localized hot spots, shock waves, and microstreaming, thereby accelerating mass transport and continuously renewing the catalyst surface. Simultaneously, VL irradiation promotes charge separation within the Ag/MFO heterointerface, while H_2_O_2_ participates in the generation of highly reactive ROS that efficiently oxidize TCH molecules [31]. The practical applicability of the optimized Ag(3wt%)/MFO photocatalyst was further evaluated through four consecutive degradation cycles under the optimized US/VL-photo-Fenton conditions (Fig. 5h). The photocatalyst maintained degradation efficiencies above 94% throughout all recycling experiments, indicating excellent catalytic durability with only negligible loss of activity. The sustained performance suggests that the Ag nanodomains remain firmly anchored on the MFO surface, preserving the structural integrity of the heterointerface during repeated operation. Furthermore, the catalyst retained its chemical stability over a broad pH range (3.0-9.0) without noticeable morphological changes or detectable metal-ion leaching, demonstrating its robustness under practical reaction conditions. These results confirm that the surface-engineered Ag(3wt%)/MFO photocatalyst possesses excellent structural stability and long-term operational reliability for repeated wastewater treatment applications. The degradation kinetics of TCH were subsequently analyzed using the pseudo-first-order kinetic model (Fig. 5i). A satisfactory linear relationship between ln(C_0_/C_t_) and irradiation time was obtained for all reaction systems, confirming that the degradation process follows pseudo-first-order kinetics. The corresponding correlation coefficient (*R^2^* = 0.984 to 0.967) further validates the applicability of the kinetic model. Among the investigated photocatalysts, Ag(3wt%)/MFO exhibited the highest apparent reaction rate together with the best linear fitting, demonstrating that Ag surface engineering effectively accelerates the US/VL-photo-Fenton degradation process by facilitating more efficient interfacial charge transfer and ROS generation. Overall, the surface-engineered Ag(3wt%)/MFO photocatalyst exhibited excellent US/VL-photo-Fenton activity toward TCH degradation. The optimized operational parameters maximized the utilization of active sites and ROS, while the synergistic coupling of acoustic cavitation, VL irradiation, H_2_O_2_ activation, and the Ag/MFO heterointerface significantly enhanced the degradation efficiency. In addition to its high catalytic activity, the photocatalyst demonstrated excellent recyclability, structural stability, and enhanced reaction kinetics, highlighting its strong potential for the efficient treatment of antibiotic-contaminated wastewater.

### 3.7 Post-Reaction Structural Stability and Reactive Species Analysis

**Fig. 6:**
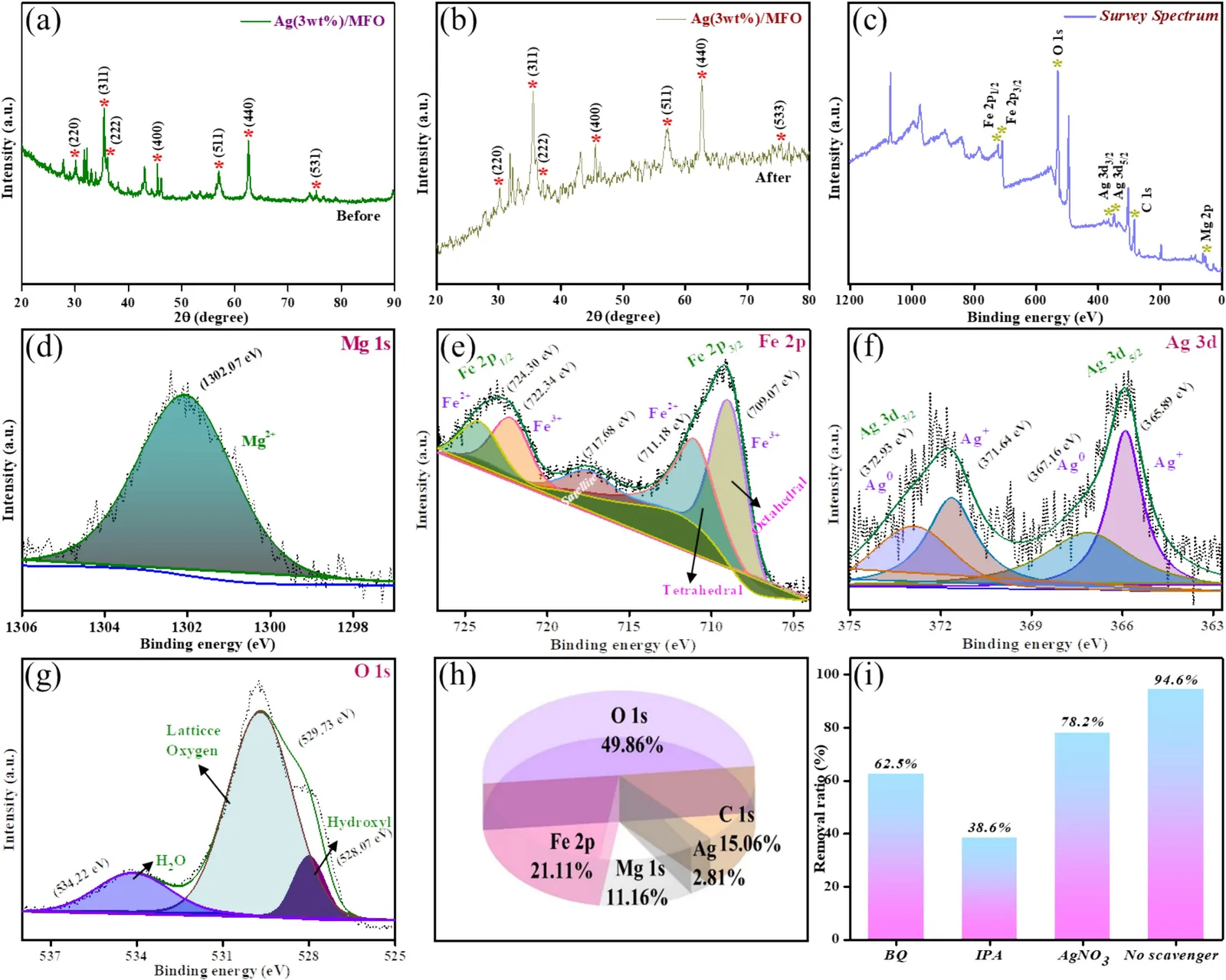
Post-reaction characterization of Ag(3 wt%)/MFO photocatalyst: (a, b) XRD patterns before and after four degradation cycles; (c) XPS survey spectrum; high-resolution XPS spectra of (d) Mg 1 s, (e) Fe 2p, (f) Ag 3d, and (g) O 1 s; (h) surface elemental composition; and (i) radical scavenging experiments during the US/VL-photo-Fenton degradation of TCH.

To evaluate the structural and chemical stability of the optimized Ag(3wt%)/MFO photocatalyst after repeated US/VL-photo-Fenton operation, post-reaction XRD and XPS, and radical scavenging analyses were performed, and the corresponding results are presented in Fig. 6(a-i). The XRD pattern of the fresh and recycled Ag(3wt%)/MFO photocatalyst after four consecutive degradation cycles is shown in Fig. 6(a,b). Before the reaction, the diffraction peaks located at 30.2^°^, 35.5^°^, 36.3^°^, 45.5^°^, 57.1^°^, 62.6^°^, and 75.4^°^ are indexed to the (220), (311), (222), (400), (511), (440), and (533) crystal planes of cubic spinel MFO. After the recycling experiments, nearly identical diffraction peaks are observed at 30.1^°^, 35.5^°^, 37.0^°^, 45.4^°^, 57.0^°^, 62.6^°^, and 75.3^°^, confirming that the crystalline spinel framework remains well preserved following repeated catalytic operation [32]. No additional diffraction peaks, measurable peak shift, or lattice distortion are detected after cycling, indicating that neither phase transformation nor secondary crystalline phases are generated during the US/VL-photo-Fenton process. Minor variations in diffraction intensity are attributed to slight differences in crystalline orientation, surface reconstruction, or sample packing during XRD measurements rather than structural degradation. These results demonstrate the excellent crystallographic stability of the Ag(3wt%)/MFO photocatalyst under repeated reaction conditions. To further examine the chemical stability of the recycled photocatalyst, post-reaction XPS analysis was carried out (Fig. 6c-h). The survey spectrum (Fig. 6c) confirms the continued presence of Mg, Fe, Ag, O, and C without the appearance of additional elemental signals, indicating that the surface composition is essentially unchanged after repeated catalytic cycles. The high-resolution Mg 1s spectrum (Fig. 6d) exhibits a single symmetric peak centered at 1302.01 eV, characteristic of Mg^2+^ species within the spinel lattice. The nearly unchanged binding energy after cycling indicates that the local chemical environment of Mg remains stable throughout the photocatalytic process. The Fe 2p spectrum (Fig. 6e) consists of the principal Fe 2p_3/2_ and Fe 2p_1/2_ peaks located at 709.07 eV and 724.30 eV, respectively, together with additional components at 711.18 eV and 722.34 eV and a shake-up satellite centered at 717.68 eV. These spectral features are characteristic of the coexistence of Fe^2−^ and Fe^3−^ species in spinel ferrites, confirming that the mixed-valence electronic structure is retained after repeated photocatalytic operation. The preservation of these redox-active iron species is favorable for maintaining continuous Fe^2+^/Fe^3+^ cycling during the US/VL-photo-Fenton reaction. The Ag 3d spectrum (Fig. 6f) reveals two pairs of spin-orbit components corresponding to oxidized and metallic silver species. The peaks located at 365.89 eV and 372.93 eV are assigned to Ag^+^ (Ag 3d_5/2_ and Ag 3d_3/2_), whereas the second pair at 367.16 eV and 371.64 eV corresponds to metallic Ag^0^. The coexistence of Ag^+^ and Ag^0^ after repeated operation indicates that the Ag/MFO heterointerface remains chemically stable. Only slight changes in binding energy are observed after cycling, suggesting minor electronic redistribution rather than degradation of the deposited Ag species. The deconvoluted O 1s spectrum (Fig. 6g) contains three components centered at 528.07, 529.73, and 534.22 eV, which are assigned to surface hydroxyl species, lattice oxygen, and adsorbed H_2_O molecules, respectively. The preservation of these oxygen environments after repeated use indicates that the surface oxygen chemistry remains largely unchanged throughout the catalytic process. The corresponding elemental composition obtained from the XPS survey analysis (Fig. 6h) shows surface atomic percentages of Mg 1s (11.16%), Fe 2p (21.11%), Ag 3d (2.81%), O 1s (49.86%), and C 1s (15.06%), further confirming that the surface composition is maintained after repeated photocatalytic operation. Overall, the post-reaction XRD and XPS results demonstrate that the Ag(3wt%)/MFO photocatalyst preserves its crystalline framework, elemental composition, oxidation states, and surface chemical environment during repeated US/VL-photo-Fenton cycles, highlighting its excellent structural durability and chemical stability under oxidation reaction conditions [6].

To identify the dominant reactive species responsible for TCH degradation, radical scavenging experiments were performed using BQ, IPA, and AgNO_3_ as selective quenchers for •O_2_^−^, •OH, and photogenerated electrons (e^−^), respectively (Fig. 6i). In addition of each scavenger resulted in a noticeable decrease in degradation efficiency, confirming the cooperative participation of multiple reactive species during the US/VL-photo-Fenton process. Among the investigated scavengers, IPA produced the most significant inhibition, reducing the degradation efficiency to 38.6%, demonstrating that •OH radicals are the predominant oxidation species responsible for TCH degradation. The presence of BQ decreased the degradation efficiency by 62.5%, indicating that •O_2_^−^ radicals also contribute to the degradation pathway but play a secondary role compared with hydroxyl radicals. In contrast, the addition of AgNO_3_ resulted in a relatively moderate decrease to 78.2%, suggesting that photogenerated electrons primarily facilitate interfacial electron transfer and promote continuous Fe^3+^/Fe^2+^ redox cycling rather than directly oxidizing TCH [33]. These results demonstrate that the excellent photocatalytic performance of Ag(3wt%)/MFO originates from the synergistic participation of •OH radicals, •O_2_^−^ radicals, and photogenerated electrons, with •OH serving as the dominant reactive species during the US/VL-photo-Fenton degradation process.

### 3.8 Proposed US/VL-Photo-Fenton Mechanism over Ag(3wt%)/MFO Photocatalyst

**Fig. 7:**
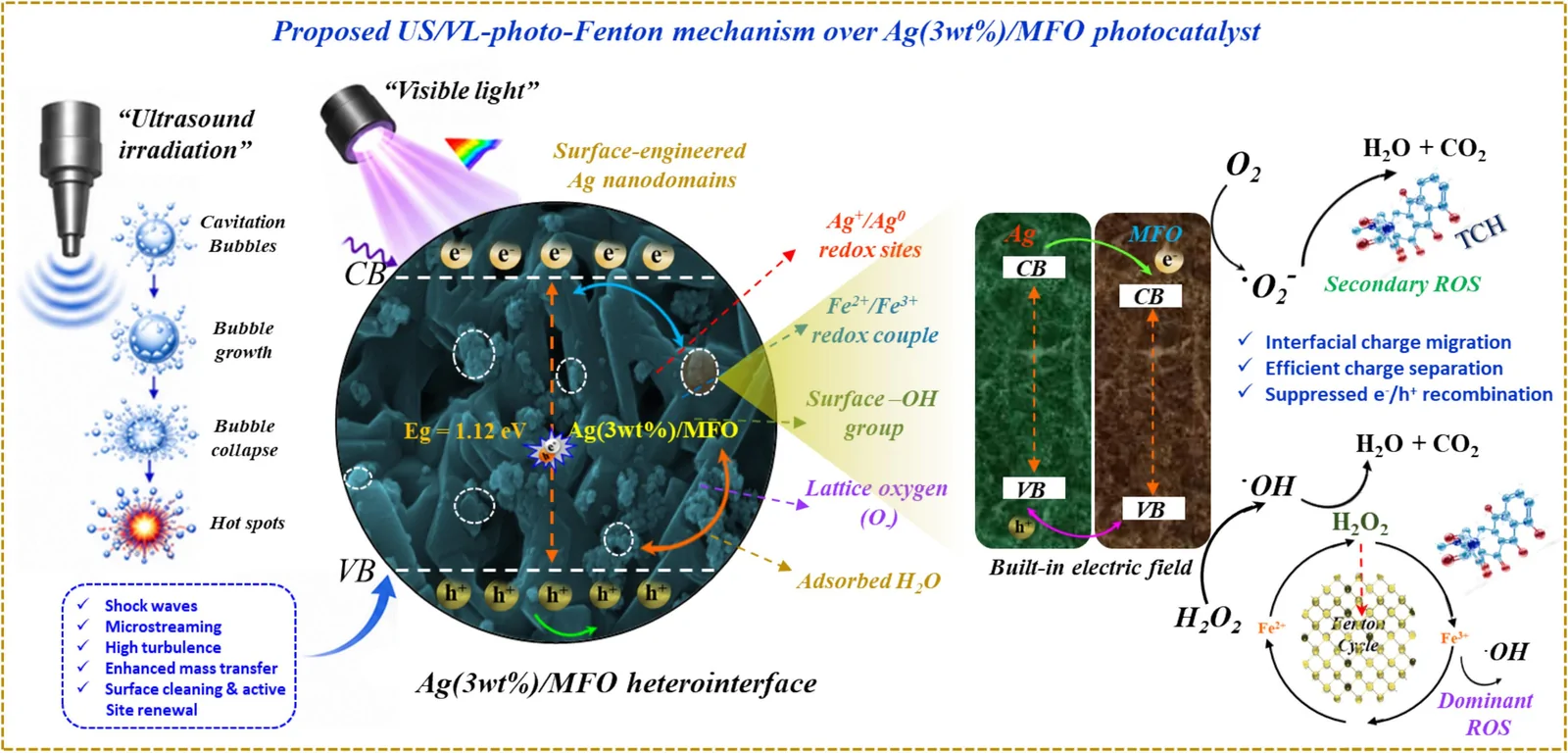
Proposed US/VL-photo-Fenton degradation mechanism over the surface-engineered Ag(3 wt%)/MFO photocatalysts, illustrating interfacial charge separation and transfer, Fe^2+^/Fe^3+^ redox cycling, H_2_O_2_ activation, ROS generation, and the mineralization of TCH.

The proposed mechanism for the US/VL-Photo-Fenton degradation of TCH over Ag(3wt%)/MFO photocatalysts is illustrated in Fig. 7. The outstanding photocatalytic performance originates from the synergistic integration of VL excitation, ultrasonic cavitation, Ag-induced interfacial charge separation, H_2_O_2_ activation, and continuous Fe^2+^/Fe^3+^ cycling, which collectively promote the efficient generation of ROS. Upon VL irradiation, the MFO semiconductor absorbs photons with energy equal to or greater than its band gap, producing photogenerated electrons in the conduction band and holes in the valence band [34]:

(2) $$Ag\left(3wt\%\right)/MFO+hv\to e_{\mathrm{CB}}^{-}+h_{\mathrm{VB}}^{+}$$

Unlike pristine MFO, the uniformly distributed Ag nanodomains establish an intimate heterointerface with the MFO surface. The interfacial architecture facilitates directional electron migration from MFO toward the Ag^-^ containing surface, while holes remain predominantly within the valence band of MFO. Consequently, the spatial separation of photogenerated charge carriers is significantly improved, suppressing electron-hole recombination and prolonging carrier lifetime under VL irradiation. The metallic Ag^0^ species serve as electron-transport pathways, whereas Ag^+^ species contribute to maintaining interfacial electronic equilibrium, thereby promoting continuous charge migration across the Ag/MFO interface.

Simultaneously, ultrasonic irradiation introduces acoustic cavitation throughout the reaction medium. The repeated formation and collapse of cavitation bubbles generate localized high-temperature and high-pressure microenvironments together with shock waves and microstreaming. These physical effects continuously renew the catalyst surface, accelerate mass transport, enhance the dispersion of H_2_O_2_ and dissolved oxygen, and improve contact between TCH molecules and catalytically active sites, thereby strengthening the overall photo-Fenton reaction [35]:

The photogenerated electrons are rapidly captured by dissolved oxygen molecules to produce superoxide radicals:

(3) $$O_{2}+e_{\mathrm{CB}}^{-}\to {\;}^{.}O_{2}^{-}$$

The generated superoxide radicals subsequently undergo proton-assisted reactions to produce hydrogen peroxide:

(4) $${\;}^{.}O_{2}^{-}+H^{+}\;\to^{.}HO_{2}$$

(5) $$2HO_{2}^{.}\to H_{2}O_{2}+O_{2}\;$$

Meanwhile, photogenerated holes oxidize surface hydroxyl groups and adsorbed water molecules to generate hydroxyl radicals:

(6) $$H_{2}O+h_{\mathrm{VB}}^{+}\to^{.}OH+H^{+}$$

In parallel, the intrinsic Fe^2+^/Fe^3+^ redox couple within the spinel MFO lattice continuously produces H_2_O_2_ through the photo-Fenton pathway:

(7) $$Fe^{2}+H_{2}O_{2}\to Fe^{3}{+}^{.}OH+OH^{-}$$

The regenerated Fe^3+^ species are subsequently reduced by photogenerated electrons, regenerating Fe^2+^ and sustaining continuous catalytic cycling:

(8) $${\text{Fe}}^{3+}+e_{\text{CB}}^{-}\to {\text{Fe}}^{2+}$$

Additional hydroxyl radicals are also produced through electron- and hole-assisted decomposition of H_2_O_2_.

(9) $$H_{2}O_{2}+e^{-}\;\to^{.}OH+OH^{-}$$

(10) $$H_{2}O_{2}+h^{+}\to^{.}OH+H^{+}$$

The radical-quenching experiments demonstrate that **^.^**OH radicals are the dominant oxidative species, while •O_2_^−^ radicals and photogenerated electrons contribute synergistically to the overall oxidation process. The abundant ROS generated through these complementary pathways attack adsorbed TCH molecules via successive oxidative reactions, ultimately yielding small oxygenated intermediates before complete mineralization into CO_2_, H_2_O, and inorganic ions:

(11) $$\mathrm{TC}H_{\;}{+}^{.}OH{/}^{.}O_{2}^{-}\to \;\text{Intermediates}\;\to {\text{CO}}_{2}+{\text{H}}_{2}\text{O}+\;\text{inorganic ions}\;\;\;\;\;$$

Overall, the exceptional US/VL-photo-Fenton activity of the Ag(3wt%)/MFO photocatalysts arises from the cooperative integration of Ag-mediated interfacial charge separation, ultrasonic cavitation, efficient H_2_O_2_ activation, and continuous Fe^2+^/Fe^3+^ redox cycling [36]. These integrated processes maximize ROS production while suppressing charge recombination, enabling rapid degradation and mineralization of TCH and demonstrating the strong potential of the developed photocatalyst for advanced wastewater remediation.

### 3.9 US/VL-Photo-Fenton Degradation Pathway of TCH

**Fig. 8:**
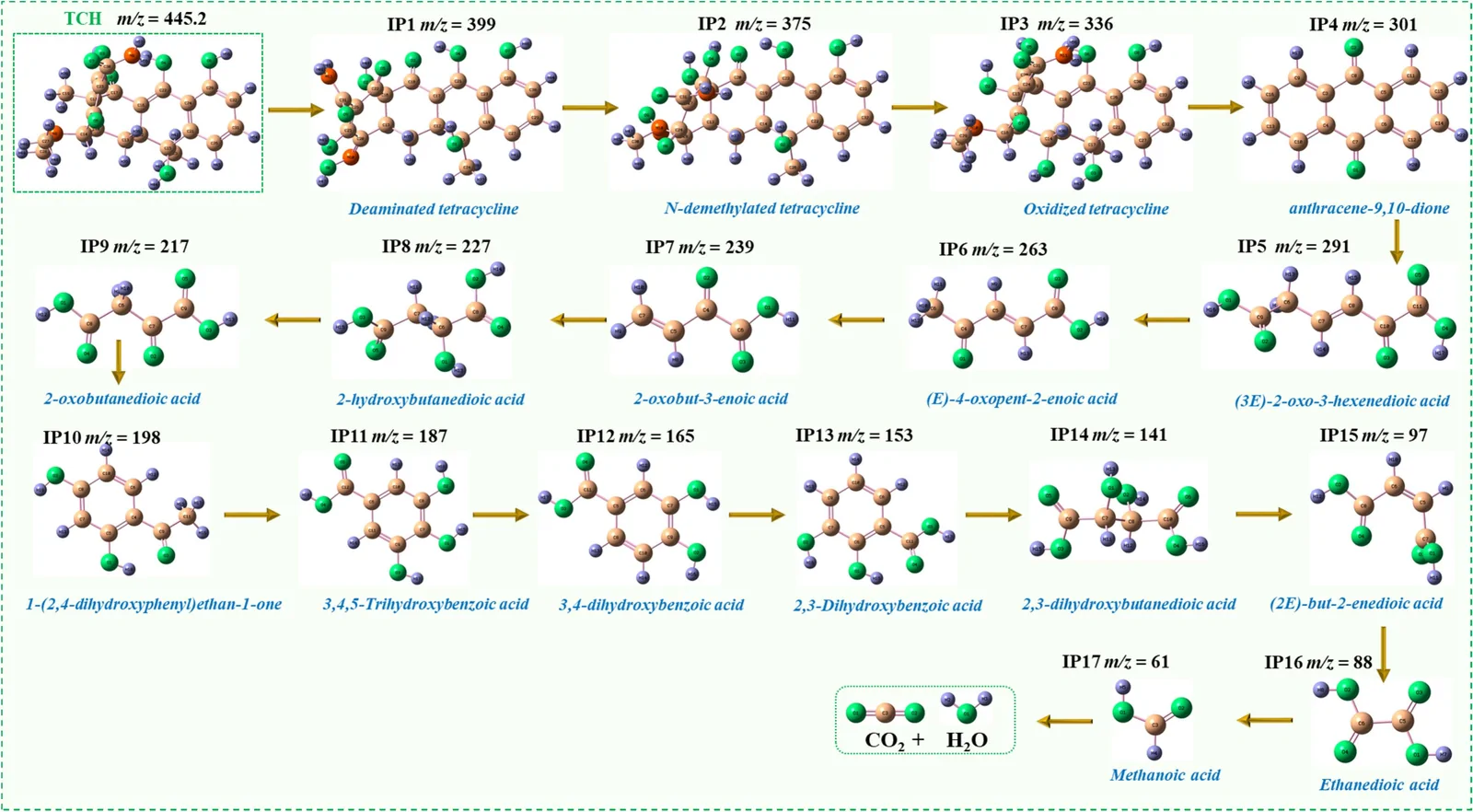
Proposed degradation pathway of TCH during the US/VL-photo-Fenton process over the Ag(3 wt%)/MFO photocatalyst, illustrating the LC-MS-identified intermediates and final mineralization products.

To elucidate the degradation pathways of TCH over the optimized Ag(3wt%)/MFO photocatalyst, the reaction intermediates formed during the US/VL-photo-Fenton process were analyzed by LC-M. The detected intermediates, together with their corresponding *m*/*z* values and proposed chemical structures, are summarized in Fig. S4 and Table S2, while the overall degradation pathways is illustrated in Fig. 8. The identified intermediates indicated that TCH degradation proceeds through a sequence of hydroxylation, deamination, N-demethylation, oxidation, aromatic ring cleavage, and progressive fragmentation reactions induced by ROS generated during the photo-Fenton process. The parent TCH molecule, detected at m/z = 445.2, initially undergoes deamination, producing the intermediate IP1 (m/z = 399). Subsequently, N-demethylation generates IP2 (m/z = 375), followed by further oxidative transformation to IP3 (m/z = 336), corresponding to an oxidized tetracycline derivative. Continued ROS attack promoted cleavage of the conjugated framework, yielding anthracene-9,10-dione (IP4, m/z = 301). Further oxidation and ring-opening reactions subsequently produce (3E)-2-oxo-3-hexenedioic acid (IP5, m/z = 291), (E)-4-oxopent-2-enoic acid (IP6, m/z = 263), and 2-oxobut-3-enoic acid (IP7, m/z = 239). Successive oxidation converts these intermediates into 2-hydroxybutanedioic acid (IP8, m/z = 227) and 2-oxobutanedioic acid (IP9, m/z = 217). Further degradation of the residual aromatic fragments results in the formation of low-molecular-weight oxygenated compounds, including 1-(2,4-dihydroxyphenyl)ethan-1-one (IP10, m/z = 198), 3,4,5-trihydroxybenzoic acid (IP11, m/z = 187), 3,4-dihydroxybenzoic acid (IP12, m/z = 165), and 2,3-dihydroxybenzoic acid (IP13, m/z = 153). Continued oxidative ring opening generates 2,3-dihydroxybutanedioic acid (IP14, m/z = 141) and (2E)-but-2-enedioic acid (IP15, m/z = 97). These short-chain carboxylic acids are subsequently transformed into ethanedioic acid (IP16, m/z = 88) and methanoic acid (IP17, m/z = 61) before complete mineralization into CO_2_ and H_2_O [37], [38]. The identified intermediates demonstrate that the degradation process is predominantly driven by ·OH radicals, together with the auxiliary contribution of •O_2_^−^ radicals, consistent with the radical-quenching experiments. These ROS continuously attack the tetracycline framework, resulting in successive oxidation, dealkylation, deamination, hydroxylation, aromatic ring opening, and fragmentation into progressively smaller oxygenated molecules, which are ultimately mineralized to environmentally benign products. Overall, the LC-MS analysis confirmed that the Ag(3wt%)/MFO-mediated US/VL-photo-Fenton system provides an efficient oxidation pathway for the complete degradation and mineralization of TCH, minimizing the persistence of stable organic intermediates and highlighting its potential for advance antibiotic wastewater remediation.

### 3.10 Density Functional Theory Insights into the Electronic Structure and Reactive Sites of TCH

**Fig. 9:**
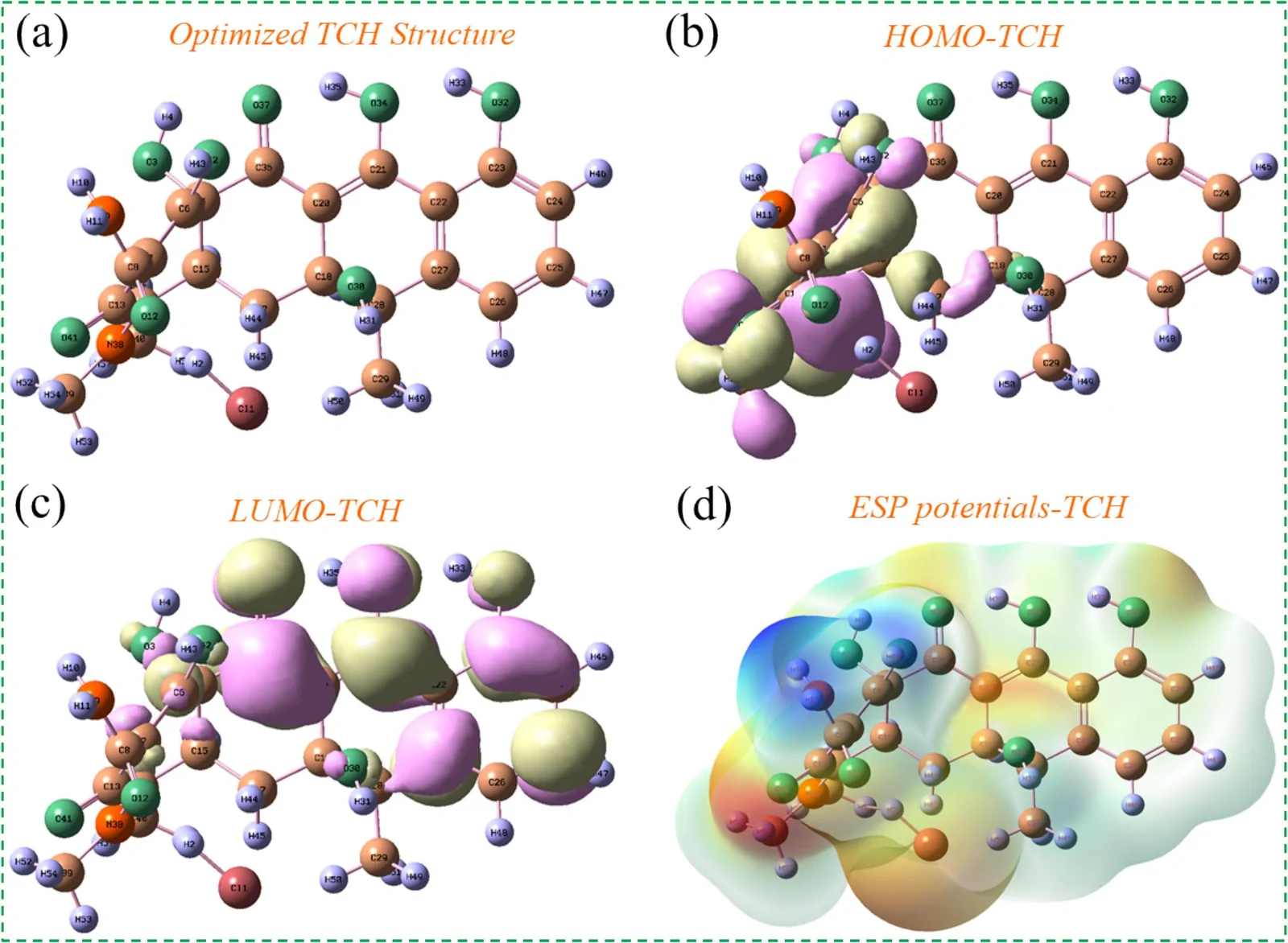
DFT analysis of the TCH molecule: (a) optimized molecular geometry, (b) HOMO distribution, (c) LUMO distribution, and (d) electrostatic potential (ESP) map illustrating the preferred reactive sites for ROS attack during the US/VL-photo-Fenton process.

To obtain molecular-level insight into the reactivity of TCH during US/VL-photo-Fenton process, DFT calculations were performed to investigate its electronic structure and identify the preferred reactive sites for oxidation. The optimized molecular geometry, frontier molecular orbitals (HOMO/LUMO), and electrostatic potential (ESP) distributions were calculated using Gaussian 16 and visualized with GaussView 6.0, as presented in Fig. 9(a-d). These calculations provide theoretical support for experimentally observed photocatalytic degradation behavior by revealing electronic characteristics governing ROS interactions with TCH [39]. As illustrated in Fig. 9a, the optimized molecular structure of TCH exhibits a non-planar tetracyclic framework containing multiple hydroxyl, carbonyl, and amide functional groups, resulting in an uneven distribution of electron density throughout the molecule. Such structural features create several chemically active regions that are susceptible to oxidative attack during the photocatalytic process. The frontier molecular orbital distributions are presented in Fig. 9(b,c). The molecule possesses a HOMO energy of –5.64 eV and a LUMO energy of –2.58 eV, corresponding to a HOMO–LUMO energy gap of 3.06 eV. The relatively small energy gap suggests that the molecule can be readily polarized under photocatalytic conditions, facilitating electron-transfer processes and enhancing its oxidation susceptibility [40]. The HOMO is predominantly localized over the phenolic hydroxyl groups, amide moiety, and adjacent conjugated aromatic rings, indicating that these electron-rich regions are the most favorable sites for electrophilic ROS attack. In contrast, the LUMO is distributed mainly across the conjugated aromatic framework, suggesting favorable electron-accepting characteristics during the oxidation process. The electrostatic potential map Fig. 9d further reveals distinct charge distributions over the TCH molecule. Regions exhibiting negative electrostatic potential are concentrated around the oxygen-containing functional groups, particularly hydroxyl and carbonyl oxygen atoms, reflecting high electron density and strong affinity toward electrophilic oxidizing species [41]. Conversely, relatively positive electrostatic potential is distributed over the aromatic carbon framework and hydrogen-rich regions, indicating comparatively electron-deficient domains. The coexistence of these electronically distinct regions facilitates selective interactions between TCH and the ROS generated during the US/VL-photo-Fenton process. Overall, the DFT calculations establish a clear relationship between the electronic structure and the degradation reactivity of TCH. The frontier molecular orbital distributions together with the ESP analysis identify the electron-rich functional groups as primary reactive centers for ROS attack, providing molecular-level evidence for the high degradation efficiency observed experimentally. These theoretical findings are consistent with the proposed photocatalytic mechanism and further support the excellent oxidation performance of the surface-engineered Ag(3wt%)/MFO photocatalyst under US/VL-photo-Fenton conditions.

## 4 Conclusion

In this work, Ag(3wt%)/MFO photocatalysts were successfully fabricated through a surface engineering strategy and systematically evaluated for the US/VL-photo-Fenton degradation of TCH. Surface modification with Ag established stable heterointerfaces that promoted interfacial charge separation, accelerated electron migration, and enhanced H_2_O_2_ activation, thereby enhancing ROS generation. Among the synthesized photocatalysts, Ag(3wt%)/MFO exhibited the highest photocatalytic activity, achieving 98.4% TCH degradation within 120 min under the optimized reaction conditions. The catalyst also demonstrated excellent structural stability and recyclability, maintaining over 94% degradation efficiency after four consecutive cycles, while post-reaction XRD and XPS analyses confirmed the preservation of its crystalline framework and surface chemical states. Radical trapping experiments identified •OH radicals as the dominant reactive species, whereas •O_2_^−^ radicals and photogenerated electrons contributed synergistically to the degradation process. Furthermore, LC-MS analysis elucidated the degradation pathway of TCH, while DFT calculations identified the electron-rich functional groups as the preferred reactive sites for ROS attack, providing molecular-level support for the proposed mechanism. The enhanced photocatalytic performance is attributed to the synergistic coupling of ultrasonic cavitation, VL excitation, Ag/MFO heterointerfaces, and Fe^2+^/Fe3+ redox cycling, collectively enhancing ROS generation and TCH degradation. The magnetic nature and good recyclability of Ag(3wt%)/MFO support its potential for catalyst recovery and repeated wastewater treatment. Overall, this study demonstrates that surface-engineered Ag(3wt%)/MFO is a highly efficient and durable US/VL-photo-Fenton photocatalyst, providing a promising platform for the sustainable remediation of antibiotic-contaminated wastewater.

## CRediT authorship contribution statement

**Velu Subash:** Writing – original draft, Methodology, Investigation, Data curation, Conceptualization. **Duraisamy Elango:** Writing – review & editing, Formal analysis, Data curation. **Palaniyappan Jayanthi:** Writing – review & editing, Supervision, Formal analysis. **Velu Manikandan:** Writing – original draft, Methodology, Data curation, Conceptualization. **Kwang Soup Song:** Writing – review & editing, Supervision, Funding acquisition, Formal analysis.

## Declaration of competing interest

The authors declare that they have no known competing financial interests or personal relationships that could have appeared to influence the work reported in this paper.

## Appendix A Supplementary data

Supplementary Data 1 Supplementary Material: Additional experimental details, characterization results, supporting figures, and tables.
