# Supplementary material for: Ultrasound-assisted visible-light photo-Fenton degradation of tetracycline over surface-engineered Ag/MgFe2O4 heterointerfaces: mechanistic and DFT insights
Source: Ultrason Sonochem. 2026 Aug 18;133:108011. doi: 10.1016/j.ultsonch.2026.108011 (PMC13524638; doi:10.1016/j.ultsonch.2026.108011)
Supplement: Supplementary file 1 [file mmc1.docx]

**Supporting Information**

**Materials and Methods**

**2.5.1 Effect of Catalyst Dosage**

The influence of catalyst dosage on the US/VL-photo-Fenton degradation of TCH was investigated using the Ag(3wt%)/MFO photocatalyst. Catalyst loadings of 20, 30, and 40 mg were separately dispersed in 100 mL of TCH solution (20 mg L^-1^) reaction systems to evaluate dosage-dependent performance. The suspensions were magnetically stirred in the dark for 30 min to establish adsorption-desorption equilibrium before the reaction. Subsequently, a predetermined volume of 30 wt% H_2_O_2_ (100 μL) was introduced, and the solution pH was adjusted to 5.8. The reaction was then carried out under simultaneous US irradiation (120 W, 37 kHz) and VL illumination (300 W Xe lamp, λ ≥ 420 nm) for 120 min. At regular 20 min intervals, 1 mL aliquots were withdrawn, centrifuged at 5000 rpm for 10 min, and the supernatant was analyzed using a UV-Vis spectrophotometer to determine the residual TCH concentration. The degradation efficiency obtained at each catalyst loading was compared to identify the optimum dosage for the subsequent experiments.

**2.5.2 Effect of Initial TCH Concentration**

The influence of the initial TCH concentration on the US/VL-photo-Fenton performance of Ag(3wt%)/MFO was investigated using aqueous solutions containing 10, 20, and 30 mg/L of TCH. A fixed amount of photocatalyst (30 mg) was dispersed in 100 mL of each solution. Before initiating the reaction, the suspension was magnetically stirred in the dark 30 min to establish adsorption-desorption equilibrium. Subsequently, 100 mL of 30 wt% H_2_O_2_ was added, and the solution pH was adjusted to 5.8. The reaction was then carried out under simultaneous US irradiation (120 W, 37 kHz) and VL illumination using a 300 W Xe lamp equipped with a λ ≥ 420 nm cutoff filter for 120 min. During the reaction, 1 mL aliquots were collected at 20 min intervals, centrifuged at 5000 rpm for 10 min, and the supernatant was analyzed by UV-Vis spectroscopy to determine the residual TCH concentration. The degradation efficiencies obtained at different initial pollutant concentrations were subsequently compared to identify the optimum operating condition.

**2.5.3 Effect of Initial Solution pH**

The effect of the initial solution pH on the US/VL-photo-Fenton degradation of TCH was investigated over pH range of 3.0-9.0 using the Ag(3wt%)/MFO photocatalyst. TCH solution (20 mg L^-1^, 100 mL) was adjusted to the desired pH values (3.0, 4.0, 5.8, 7.0, and 9.0) using 0.1 m HCl and 0.1 NaOH. Subsequently, 30 mg of photocatalyst was dispersed in the solution, and the suspension was magnetically stirred in the dark for 30 min to establish adsorption-desorption equilibrium. Therefore, 100 μL of 30 wt% H_2_O was introduced, and the reaction was initiated under simultaneous ultrasonic irradiation (120 W, 37 kHz) and VL illumination from a 300 W Xe lamp (λ ≥ 420 nm) for 120 min. During the reaction, 1 mL aliquots were collected at 20 min intervals, centrifuged at 5000 rpm for 10 min, and the supernatant was analyzed by UV-vis spectroscopy to determine the residual TCH concentration. The degradation efficiencies obtained at different pH values were compared to identify the optimum reaction pH.

**2.5.4 Effect of H_2_O_2_ Dosage**

The influence of H_2_O_2_ dosage on the US/VL-photo-Fenton degradation of TCH was investigated using the Ag(3wt%)/MFO photocatalyst. A fixed amount of photocatalyst (30 mg) was dispersed in 100 mL of TCH solution (20 mg L^-1^) at an initial pH of 5.8. The suspension was magnetically stirred in the dark for 30 min to stablish adsorption-desorption equilibrium. Subsequently, different volumes of 30 wt% H_2_O_2_ (50, 100, and 150 μL) were added to evaluate their influence on the degradation efficiency. The reaction was carried out under simultaneous ultrasonic irradiation (120 W, 37 kHz) and VL illumination using 300 W Xe lamp equipped with a λ ≥ 420 nm cutoff filter for 120 min. During the reaction, 1 mL aliquots were withdrawn at 20 min intervals, centrifuged at 5000 rpm for 10 min, and the supernatant was analyzed by UV-Vis spectroscopy to determine the residual TCH concentration. The degradation efficiencies obtained at different H_2_O_2_ dosages were compared to determine the optimum oxidant concentration for the US/VL-photo-Fenton process.

**2.7 Identification of Reactive Species by Scavenger Experiments**

The dominant reactive species involved in the US/VL-photo-Fenton degradation of TCH were identified using selective radical scavenging experiments. A 100 mL aqueous solution containing 20 mg L^-1^ of TCH was mixed with 30 mg of Ag(3wt%)/MFO photocatalyst and magnetically stirred in the dark for 30 min to establish adsorption-desorption equilibrium. Subsequently, 100 μL of 30 wt% H_2_O_2_ was added, and the solution pH was adjusted to 5.8. To selectively quench the reactive species, isopropanol (IPA, 1 mM), benzoquinone (BQ, 1 mM), and silver nitrate (AgNO_3,_ 1 mM) were separately introduced as scavengers for •OH radicals, •O₂⁻, radicals, and photogenerated electrons (e⁻), respectively. A control experiment performed without any scavenger was used for comparison. The reaction was conducted under simultaneous ultrasonic irradiation (120 W, 37 kHz) and VL illumination using 300 W Xe lamp equipped with a λ ≥ 420 nm cutoff filter for 120 min. During the reaction, 1 mL aliquots were withdrawn at 20 min intervals, centrifuged at 5000 rpm for 10 min, and the supernatant was analyzed by UV-Vis spectroscopy to determine the residual TCH concentration. The changes in degradation efficiency in the presence scavengers were used to identify the predominant reactive species responsible for the US/VL-photo-Fenton process.

**2.8 Density Functional Theory (DFT) Calculations**

Density functional theory (DFT) calculations were performed to investigate the electronic properties and reactive sites of the TCH molecule. All calculations were out using *Gaussian 09*, and the optimized molecular structures together with the frontier molecular orbitals and electrostatic potential (ESP) maps were visualized using *Gauss View 6.0*. The molecular geometry was fully optimized employing the B3LYP exchange-correlation functional with the 6-31G(d,p) basis set. Vibrational frequency calculations were subsequently performed to confirm that the optimized structure corresponded to a true energy minimum without imaginary frequencies. The optimized geometry was used to determine the energies and spatial distributions of the highest occupied molecular orbital (HOMO) and lowest unoccupied molecular orbital (LUMO). The HOMO-LUMO energy gap was evaluated to assess the electronic stability and chemical reactivity of the TCH molecule. In addition, electrostatic potential (ESP) mapping was performed to identify electron-rich and electron-deficient regions that are susceptible to ROS attack during US/VL-photo-Fenton process. The computational results were subsequently correlated with the experimental degradation behavior and LC-MS analysis to provide molecular-level insight into the degradation mechanism.

**Fig. S1.** FESEM-EDS spectra and corresponding elemental mapping images of pristine MFO (a-c), Zr(3wt%)/MFO (d-f), Co(3wt%)/MFO (g-i), and Ag(3wt%)/MFO (j-l) photocatalysts.

**Fig. S2.** BJH pore-size distribution curves of (a) pristine MFO, (b) Zr(3wt%)/MFO, (c) Co(3wt%)/MFO, and (d) Ag(3wt%)/MFO photocatalysts.

**Fig. S3.** Photoluminescence spectra of pristine MFO, Zr(3wt%)/MFO, Co(3wt%)/MFO, and Ag(3wt%)/MFO photocatalysts.

**Fig. S4.** LC-MS spectrum of the degradation intermediates detected during the US/VL-photo-Fenton degradation of TCH over the Ag(3wt%)/MFO photocatalyst.

**Table S1.** Comparative of the photocatalytic degradation performances of Ag(3wt%)/MFO with previously reported photocatalysts for TCH removal under US/VL-photo-Fenton conditions.

| **Photocatalyst** | **Structural**  **Features** | **Targeted  Pollutant** | **Degradation**  **Efficiency** | **Light Source** | **Ultrasound Frequency** | **Catalyst**  **Dose (g/L)** | **Time**  **(min)** | **H_2_O_2_**  **Applied** | **DFT  Analysis** | **Reference** |
| --- | --- | --- | --- | --- | --- | --- | --- | --- | --- | --- |
| Ag_6_Si_2_O_7_/SmFeO_3_ | Coral-like | Ciprofloxacin | 94.9 | Visible | 40 kHz | 0.6 | 60 | Yes | No | Yun et al., 2023 |
| CoFe_2_O_4_@TiO_2_@rGO | Spherical | Tetracycline | 92 | Visible | 40 kHz | 0.03 | 90 | No | Yes | Nimshi et al., 2023 |
| CoFe_2_O_4_@TiO_2_@rGO | Spherical | Ciprofloxacin | 84 | Visible | 40 kHz | 0.03 | 90 | No | Yes |  |
| NiFe-LDH/rGO | Micropheres | Moxifloxacin | 90.40 | Visible | 36 kHz | 1 | 60 | No | No | Khataee et al., 2019 |
| CuFe_2_O_4_/montmorillonite | Lamellar  structure | Ciprofloxacin | 93.1 | Uv | 50 kHz | 0.6 | 60 | No | No | Al-Musawi |
| rGO/Ag_2_CO_3_ | Sheet-like | Tetracycline | 97.3 | Visible | 33 kHz | 0.3 | 60 | No | No | Reheman et al., 2019 |
| Cu-MOF/ZnWO_4_ | Flower-like | Tetracycline | 98 | Visible | 40 kHz | 0.02 | 60 | Yes | No | Jeyaprakash et al., 2023 |
| Bi_2_MoO_6_/FeVO_4_ | Porous- nanorod | Ciprofloxacin | 98.2 | Visible | 40 kHz | 0.5 | 60 | Yes | Yes | Fan et al., 2022 |
| CdS/ZnO | Sheet-like | Tetracycline | 84 | Visible | 50 kHz | 0.03 | 90 | No | No | Esmaeili et al., 2023 |
| SnO_2_ | Nanoflakes | Tetracycline | 88.8 | Visible | 40 kHz | 3 | 163 | No | No | Yashas et al., 2020 |
| MoS_2_/C | Urchin-like | Levofloxacin | 100 | Visible | 40 kHz | 0.01 | 180 | No | No | Zeng et al., 2019 |
| g-C_3_N_4_ | Sheet-like | Tetracycline | 96 | Visible | 40 kHz | 0.01 | 60 | No | Yes | Preeyanghaa et al., 2023 |
| PM-TiO_2_-Pd | Sheet-like | Ciprofloxacin | 79.44 | Visible | 40 kHz | 0.5 | 120 | No | No | Motlagh et al., 2021 |
| **Ag(3wt%)/MFO** | **Heterointerface** | **Tetracycline** | **98.4** | **Visible** | **40 kHz** | **0.3** | **120** | **Yes** | **Yes** | **Present study** |

**Reference**

K. Yun, K. Saravanakumar, G. Jagan, Y. Yea, Y. Yoon, C.M. Park. Fabrication of highly effective Ag_6_Si_2_O_7_/SmFeO_3_ heterojunction with synergistically enhanced sonophotocatalytic degradation of ciprofloxacin and production of H_2_O_2_: Influencing factors and degradation mechanism. Chem. Eng. J., 468 (2023), 143491.

R.E. Nimshi, J.J. Vijaya, L.J. Kennedy, P.S. Selvamani, M. Bououdina, P.J. Sophia. Effective microwave-assisted synthesis of CoFe_2_O_4_@TiO_2_@rGO ternary nanocomposites for the synergic sonophotocatalytic degradation of tetracycline and c antibiotics. Ceram. Int., 49 (2023), pp. 13762-13773

A. Khataee, T. Sadeghi Rad, S. Nikzat, A. Hassani, M.H. Aslan, M. Kobya, E. Demirbaş. Fabrication of NiFe layered double hydroxide/reduced graphene oxide (NiFe-LDH/rGO) nanocomposite with enhanced sonophotocatalytic activity for the degradation of moxifloxacin. Chem. Eng. J., 375 (2019), 122102

T.J. Al-Musawi, N. Mengelizadeh, K. Sathishkumar, S. Mohebi, D. Balarak. Preparation of CuFe_2_O_4_/montmorillonite nanocomposite and explaining its performance in the sonophotocatalytic degradation process for ciprofloxacin. Colloid Interface Sci. Commun., 45 (2021), 100532

A. Reheman, K. Kadeer, K. Okitsu, M. Halidan, Y. Tursun, T. Dilinuer, A. Abulikemu. Facile photo-ultrasonic assisted reduction for preparation of rGO/Ag_2_CO_3_ nanocomposites with enhanced photocatalytic oxidation activity for tetracycline. Ultrason. Sonochem. 51 (2019), pp. 166-177.

J. S. Jeyaprakash, M.Rajamani, C. L. Bianchi, M.Ashokkumar, B. Neppolian. Highly efficient ultrasound-driven Cu-MOF/ZnWO_4_ heterostructure: An efficient visible-light photocatalyst with robust stability for complete degradation of tetracycline. Ultrasonics Sonochemistry, 100, (2023), 106624.

G. Fan, C. Cai, S. Yang, B. Du, J. Luo, Y. Chen, X. Lin, X. Li, Y. Wang. Sonophotocatalytic degradation of ciprofloxacin by Bi_2_MoO_6_/FeVO_4_ heterojunction: Insights into performance, mechanism and pathway. Sep. Purif. Technol., 303 (2022), 122251.

A. Esmaeili, S. Pourranjabar Hasan Kiadeh, A. Ebrahimian Pirbazari, F. Esmaeili Khalil Saraei, A. Ebrahimian Pirbazari, A. Derakhshesh, F.-S. Tabatabai-Yazdi, CdS nanocrystallites sensitized ZnO nanosheets for visible light-induced sonophotocatalytic/photocatalytic degradation of tetracycline: From experimental results to a generalized model based on machine learning methods, Chemosphere. 332 (2023) 138852.

S.R. Yashas, H.P. Shivaraju, T. Thinley, K.S. Pushparaj, A. Maleki, B. Shahmoradi. Facile synthesis of SnO_2_ 2D nanoflakes for ultrasound-assisted photodegradation of tetracycline hydrochloride. Int. J. Environ. Sci. Technol., 17 (2020), pp. 2593-2604.

L. Zeng, S. Li, X. Li, J. Li, S. Fan, X. Chen, Z. Yin, M. Tadé, S. Liu. Visible-light-driven sonophotocatalysis and peroxymonosulfate activation over 3D urchin-like MoS_2_/C nanoparticles for accelerating levofloxacin elimination: Optimization and kinetic study. Chem. Eng. J., 378 (2019), Article 122039.

M. Preeyanghaa, E.S. Erakulan, R. Thapa, M. Ashokkumar, B. Neppolian. Scrutinizing the role of tunable carbon vacancies in g-C_3_N_4_ nanosheets for efficient sonophotocatalytic degradation of Tetracycline in diverse water matrices: Experimental study and theoretical calculation. Chem. Eng. J., 452 (2023), Article 139437.

P.Y. Motlagh, S. Akay, B. Kayan, A. Khataee. Ultrasonic assisted photocatalytic process for degradation of ciprofloxacin using TiO_2_-Pd nanocomposite immobilized on pumice stone. J. Ind. Eng. Chem., 104 (2021), pp. 582-591.

**Table S2.** Proposed LC-MS-identified intermediates of TCH, together with their corresponding mass-to-charge (*m/z*) values and proposed chemical structures.

| **Intermediates** | **m/z** | **Structure name** | **IUPAC name** | **3D structure** |
| --- | --- | --- | --- | --- |
| TCH | 445.2 | Tetracycline Hydrochloride | (4S,4aS,5aS,6S,12aS)-4-(dimethylamino)-1,4,4a,5,5a,6,11,12a-octahydro-3,6,10,12,12a-pentahydroxy-6-methyl-1,11-dioxo-2- naphthacenecarboxamide |  |
| IP1 | 339 | Deaminated tetracycline | 4S,4aS,5aS,6S,12aR)-4-(dimethylamino)-1,6,10,11,12a-pentahydroxy-6-methyl-3,12-dioxo-4,4a,5,5a-tetrahydrotetracene-2-carboxylic acid |  |
| IP2 | 375 | N-demethylated tetracycline | (4Z,4aS,5aS,6S,12aS)-3,6,10,12,12a-pentahydroxy-4-hydroxyimino-6-methyl-1,11-dioxo-1,4,4a,5,5a,6,12,12a-octahydrotetracene-2-carboxamide |  |
| IP3 | 336 | Oxidized tetracycline intermediate | (4S,4aR,5S,5aR,6S,12aS)-4-(Dimethylamino)-3,5,6,10,11,12a-hexahydroxy-6-methyl-1,12-dioxo-1,4,4a,5,5a,6,12,12a-octahydrotetracene-2-carboxamide |  |
| IP4 | 301 | Ring-contracted intermediate | anthracene-9,10-dione |  |
| IP5 | 291 | Hydroxylated aromatic fragment | (3E)-2-oxo-3-hexenedioic acid |  |
| IP6 | 263 | Ring-opened diketone | (E)-4-oxopent-2-enoic acid |  |
| IP7 | 239 | Unsaturated keto acid | 2-oxobut-3-enoic acid |  |
| IP8 | 227 | Dicarboxylic acid intermediate | 2-hydroxybutanedioic acid |  |
| IP9 | 217 | Short-chain oxygenated acid | 2-oxobutanedioic acid |  |
| IP10 | 198 | Oxygenated aromatic fragment | 1-(2,4-dihydroxyphenyl)ethan-1-one |  |
| IP11 | 187 | Polyhydroxylated aromatic compound | 3,4,5-Trihydroxybenzoic acid |  |
| IP12 | 165 | Catechol-type intermediate | 3,4-dihydroxybenzoic acid |  |
| IP13 | 153 | Hydroxybenzoic acid | 2,3-Dihydroxybenzoic acid |  |
| IP14 | 141 | Small dicarboxylic acid | 2,3-dihydroxybutanedioic acid |  |
| IP15 | 97 | Maleic acid-type intermediate | (2E)-but-2-enedioic acid |  |
| IP16 | 88 | Oxalic acid | Ethanedioic acid |  |
| IP17 | 61 | Formic acid / carbonate intermediate | Methanoic acid |  |
